# Rhinoclactones A-E, Resorcylic Acid Analogs from Desert Plant Endophytic Fungus *Rhinocladiella similis*

**DOI:** 10.3390/molecules24071405

**Published:** 2019-04-10

**Authors:** Luying Li, Xiaoyan Zhang, Xiangmei Tan, Bingda Sun, Bin Wu, Meng Yu, Tao Zhang, Yonggang Zhang, Gang Ding

**Affiliations:** 1 Institute of Medicinal Plant Development, Chinese Academy of Medical Sciences and Peking Union Medical College, Beijing 100193, China; luyingli868@163.com (L.L.); zxy009018@163.com (X.Z.); xiangmei_t129@163.com (X.T.); myu@implad.ac.cn (M.Y.); zt830423@163.com (T.Z.); 2 Institute of Microbiology, Chinese Academy of Sciences, Beijing 100101, China; sunbd@im.ac.cn; 3 Biology Institute, Qilu University of Technology (Shandong Academy of Sciences), Jinan 250353, China; zhangygcq@163.com

**Keywords:** desert plant, *Agriophyllum squarrosum*, endophytic fungus, *Rhinocladiella similis*, rhinoclactones, cytotoxic activities

## Abstract

Seven resorcylic acid lactones (RALs) including five new analog rhinoclactones, A–E (**1**, **2**, **4**–**6**), were isolated from an endophytic fungus *Rhinocladiella similis* in the plant *Agriophyllum squarrosum* collected from the Tengger Desert of the Ningxia Province, China. The structures of these new compounds were determined by HR-ESI-MS (High Resolution Electrospray Ionization Mass Spectrometry), NMR data, modified Mosher’s method, and X-ray diffraction experiments. All compounds isolated from this fungus possessed the 16-OMe/14-OH, not the common 16-OH/14-OH or 16-OH/14-OMe groups on the aromatic ring, which are rarely found in nature. Compound **7** displayed cytotoxic activities against HCT116 and HeLa cancer cell lines. The possible biosynthesis of **1**–**7** is suggested, and the potential ecological roles of these fungal secondary metabolites is discussed.

## 1. Introduction

**Figure 1:**
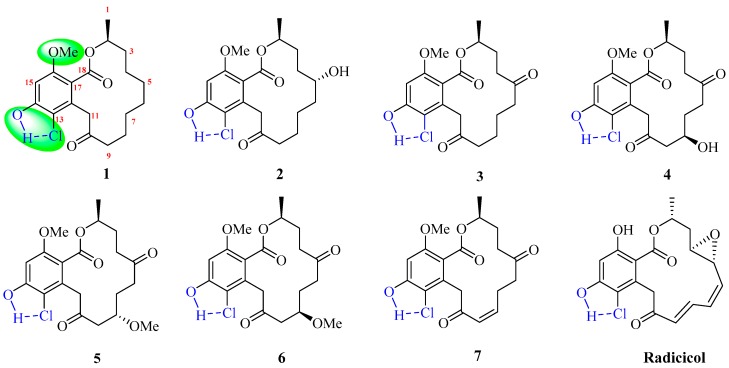
Structures of compounds **1**–**7**.

Resorcylic acid lactones (RALs) are fungal polyketides constructed by a 1, 3-benzenediol moiety fused with a macrocyclic lactone ring. The different degree of oxidation, unsaturation, and rearrangement on the macrolide ring together with methylation and chlorination on the aromatic ring diversify this group of natural products [1,2,3,4]. Resorcylic lactone analogs (such as radicicol) also display a wide range of biological activities, including inhibition of Hsp90 and kinases, antimicrobial, cytotoxic and anti-inflammatory effects [1,5,6,7,8]. Recently, our lab initiated chemical investigation of endophytes collected from desert, arid and grassland areas of West China [9,10,11,12,13,14,15]. In order to further mine new/novel and bioactive secondary metabolites from this special member of fungi, seven RAL analogs (**1**–**7**), including five new varieties (**1**, **2**, **4**–**6**) with different modifications on the macrolactone ring and phenolic ring, were isolated from an endophytic fungus *Rhinocladiella similis* from the plant *Agriophyllum squarrosum* (L.) Moq. (Amaranthaceae), collected from the Tengger Desert of the Ningxia Province, China (Figure 1). In this article, the structures of these new compounds (**1**, **2**, **4**–**6**), including the relative and absolute configurations, were established by NMR data, X-ray diffraction experiments, and chemical reactions. Bioactive evaluations of cytotoxicity, and the possible biogenetic pathways of **1**–**7** are presented, and the potentially ecological roles of this endophytic fungus are discussed.

## 2. Results and Discussion

**Figure 2:**
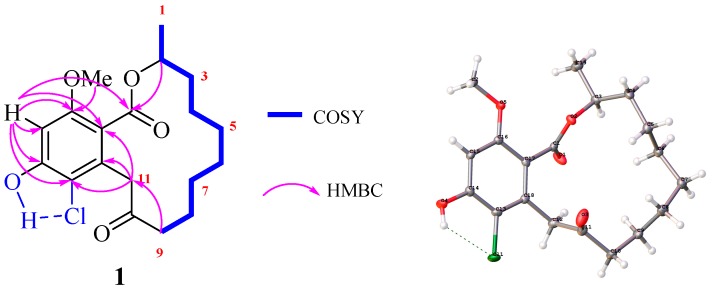
Key 2D NMR correlations and X-ray structure of **1**.

The molecular formula of rhinoclactone A (**1**) was determined to be C_1__9_H_25_ClO_5_ on the basis of HR-ESI-MS (*m*/*z* 391.1289 [M + Na]^+^, Calcd 391.1288) with seven degrees of unsaturation. The ^1^H, ^13^C, and HSQC NMR data of **1** (Table 1) revealed the presence of two methyls (one methoxyl), eight methylenes, one oxymethine unit, six aromatic carbons, and two carbonyl carbons (one ester carboxyl). These data accounted for all the ^1^H (except for one free hydroxyl group) and ^13^C-NMR resonances, suggesting two rings present in Structure **1**. A detailed comparison of the NMR and MS data of **1** with those of 8, 9-dihyrogreensporone C implied their similar structural features with the exception of the extra chloride atom and missing aromatic proton (H-13) in **1** [16]. Considering other known chloro-RAL analogs isolated from this fungus, the remaining chloride atom was suggested to be present at C-13. The ^1^H and ^13^C signals of **1** were characterized by 2D NMR experiments. Fortunately, the suitable crystal of **1** was obtained, which gave its absolute configuration as 2*S,* determined by a single crystal X-ray diffraction experiment (Figure 2). The X-ray diffraction experiments of **1** and 14-*O*-(4-bromobenzoyl)-8,9-dihydrogreensporone C revealed that the intramolecular hydrogen bond was formed between 14-OH and 13-Cl [16].

**Figure 3:**
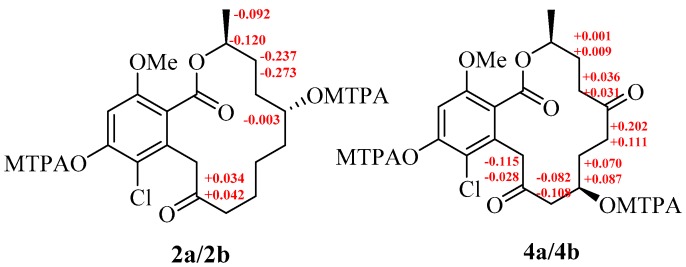
Δ*δ* values (in ppm) = *δ_S_* − *δ_R_* for (*R*)- and (*S*)-OMTPA diesters (**2a/2b** and **4a/4b**).

Rhinoclactone B (**2**) had the molecular formula as C_19_H_25_ClO_6_ on the basis of HR-ESI-MS (*m*/*z* 407.1233 [M + Na]^+^, Calcd 407.1237). The 2D NMR spectra confirmed that **2** possessed the same planar structure as that of 8, 9-dihyrogreensporone D [16]. The NMR data in these two compounds were the same, except that the chemical shift values of H/C-5 differed significantly, implying that C-5 had the opposite configuration compared with that of 8, 9-dihyrogreensporone D. Considering the absolute confirmation of C-5 in 8, 9-dihyrogreensporone D as *S*, the stereochemistry of C-5 in **2** was suggested to be *R*. This conclusion was further supported by the modified Mosher’s method (Figure 3) [17]. Three MeO groups were observed in the ^1^H-NMR spectra of products, implying that both 5-OH and 14-OH reacted with Mosher’s acyl chloride. A comparison of the ^1^H-NMR spectrum of products with that of **2** revealed that the chemical shift value of H-5 was significantly down-fielded. The difference in chemical shifts (Δ*δ* = *δ_S_* − *δ_R_*) for **2a** ((*S*)-OMTPA) and **2b** ((*R*)-OMTPA) was calculated and the absolute configuration of C-5 was assigned to 5*R* (Figure 3).

Compound (**3**) was identified to be 8, 9-dihyrogreensporone A based on HR-ESI-MS and NMR data [16]. The molecular formula of rhinoclactone C (**4**) was determined to be C_1__9_H_23_ClO_7_ on the basis of HR-ESI-MS (*m*/*z* 421.1031 [M + Na]^+^, Calcd 421.1030). A comparison of the molecular formula with that of **3** displayed that **4** had one more hydroxyl group. The peak shape of H-9a (*δ*_H_ = 2.63, *J* = 14.0, 3.5 Hz) and H-9b (*δ*_H_ = 2.33, *J* = 14.0, 9.5 Hz) was observed to be a doublet of doublets (dd), respectively, revealing that a methine unit must be connected with C-9, and this suggested that the additional hydroxyl group must have been at C-8. The conclusion was further supported by the HMBC (Heteronuclear Multiple Bond Correlation) correlation from the oxymethine (*δ*_H_ = 3.82) to C-10. The stereo-center of C-8 in **4** was determined by the modified Mosher’s method, the difference in chemical shifts (Δ*δ* = *δ_S_* − *δ_R_*) for **4a** ((*S*)-OMTPA) and **4b** ((*R*)-OMTPA) was calculated and the absolute configuration of C-8 was assigned to *R* (Figure 3).

The HR-ESI-MS (*m*/*z* 435.1187 [M + Na]^+^, Calcd 435.1187) gave the molecular formula of rhinoclactone D (**5**) to be C_20_H_25_ClO_7_ with 14 Dalton more than that of **4**. The ^1^H and ^13^C-NMR spectra revealed one more methoxyl group present in **5**. Considering the intramolecular hydrogen bond between 14-OH and 13-Cl, it was suggested that this methoxyl should be connected at C-8 not C-14, and this was confirmed by the HMBC correlation from the peak of the methyl (*δ*_H_ = 3.56) to C-8. The chemical shift values and coupling constants of H-9a and H-9b were *δ*_H_ = 3.00 (*J* = 8.0, 14.5 Hz) and *δ*_H_ = 2.52 (*J* = 1.5, 14.5 Hz), respectively, different from those of **4** (H-9a, *δ*_H_ = 2.63, *J* = 3.5, 14.0 Hz; H-9b, *δ*_H_ = 2.33, *J* = 9.5, 14.0 Hz), which implied that the configuration at C-8 in **5** was opposite to that found in **4**. The planar structure of rhinoclactone E (**6**) was determined to be the same as that of **5** on the basis of HR-ESI-MS and NMR data. The chemical shift values and coupling constants of H-9a (*δ*_H_ = 2.68, *J* = 3.5, 14.5 Hz), and H-9b (*δ*_H_ = 2.21, *J* = 9.0, 14.5 Hz) were similar to those of **4**, but different to those of **5**, implying that the configuration of C-5 in **6** was similar to that in **4**.

Compound **7** possessed the same planar structure as that of greensporone A according to the NMR data analysis [16].

Based on a similar biosynthetic pathway, the stereochemistry of C-2 in compounds (**2**, **4**–**6**) was suggested to be the same as that found in **1**, which was determined by X-ray diffraction experiment, and the known compounds **3** and **7** also possessed the same absolute configuration at C-2 as that of new analogs **1**, **2**, and **4**–**6**.

RALs are an important member of polyketides isolated from diverse fungi inhabiting niches. All compounds (**1**–**7**) isolated from *R. similis* possessed unique 16-OMe/14-OH, not the common 16-OH/14-OH or 16-OH/14-OMe groups on the aromatic ring, and all compounds (**1**–**7**) contained a chloride atom at C-13, implying that a halogenase should be contained in the biosynthetic gene cluster of these secondary metabolites [18]. The possible biosynthetic pathway of **1**–**7** was suggested as depicted in Figure S8.1 (Supplementary Materials).

Compounds **1**–**7** were evaluated for cytotoxic activities against HCT116, MCF7 and HeLa cancer cell lines, whereas only compound (**7**) displayed weak biological activities against HCT116 and HeLa cancer cell lines with IC_50_ values of 43.51 ± 1.04, 40.61 ± 0.32 μM, respectively, compared with the positive control *cis*-platinum (IC_50_ value of 11.36 ± 0.42, 3.54 ± 0.12 μM, respectively).

Heat shock protein 90 (Hsp90) is an ATP-dependent chaperone and a vital drug target related to a wide range of pathologies from oncology to neurodegenerative and other diseases [4]. The RAL analog radicicol displayed significantly inhibitory activities against Hsp90 with IC_50_ values lower than 20 nM. Structure–activity relationship studies revealed that 14,16-hydroxyls and the ester carbonyl (at C-18) in RAL structures play a crucial role in the inhibition of Hsp90 [4]. This might explain non or low-cytotoxic activities of compounds **1**–**7** against different cancer cell lines due to the absence of the free hydroxyl group at C-16 by precluding their binding with Hsp90.

Actually, 14-membered ring RALs with a 16-OMe group do not often occur in nature, and, to date, only three rare analogs have been found. This raised a very interesting question: Why did the endophytic fungus *Rhinocladiella similis* biosynthesize 14-membered ring RAL analogs (**1**–**7**) with the 16-OMe group? We postulated that methylation of 16-OH in 14-membered ring RALs might reduce cell cytotoxicity against the host plant cell to ensure that the endophytic fungus is not kicked out of the host plant’s immunologic system. In addition, all compounds **1**–**7** possessed a unique chloride atom at C-13, something that does not often occur in terrestrial fungi, though there were some analogs isolated from different fungi [4]. However, it should be taken into account that the host plant *A. squarrosum* grows in desert areas and has higher saline stress than that of other plants. It raised another interesting question: Might this endophytic fungus help its host defense of saline stress by metabolizing redundant chloride present in the desert soil to form chlorinated secondary metabolites (**1**–**7**), and thus help its host adapt to adversely abiotic environments? Clearly, more evidence is needed to elucidate the ecological roles of compounds **1**–**7**.

## 3. Experimental Section

### 3.1. General Experimental Procedures

Optical rotations were taken on a Perkin-Elmer 241 polarimeter (PerkinElmer, Waltham, MA, USA). The UV spectra were recorded on a Thermo Genesys-10S UV-Vis spectrometer (Shimadzu, Kyoto, Japan). The IR spectra were measured using a Nicolet IS5FT-IR spectrophotometer (Shimadzu, Kyoto, Japan). The 1D and 2D NMR spectra were measured in Acetone-*d*_6_ (*δ*_H_ 2.09/*δ*_C_ 206.0) on a Bruker 600 spectrometer (^1^H: 600MHz, ^13^C: 150MHz) (Bruker, Rheinstetten, Germany). HR-ESI-MS were obtained using a TOF-ESI-MS (Time of Flight Electrospray Ionization Mass Spectrometry) (Waters Synapt G2, Milford, American). Semi-preparative HPLC separation was performed on a Shimadzu LC-6AD instrument packed with a YMC-Pack ODS-A column (5 μm, 250 mm × 10 mm). Sephadex LH-20 was purchased from Pharmacia Biotech (Uppsala, Sweden). Silica gel (200–300 mesh) for column chromatography was produced by Qingdao Marine Chemical Factory, Qingdao, China.

### 3.2. Fungal Material

The strain of *R. similis* (CGMCC3.18889) was isolated from asymptomatic leaf tissue of *A. squarrosum* and was identified by morphological examination and analysis of internal transcribed spacer (ITS) of ribosomal RNA (GenBank accession No. MF511725). The fungus was grown on potato dextrose agar plates at 25 °C for 7 days. Then the fresh mycelium was inoculated into an autoclaving sterilized solid medium with a formula of rice (60.0 g) and distilled water (80 mL) in Fernbach flasks (500 mL) for further fermentation at 25 °C for 40 days.

### 3.3. Extraction and Isolation

The fermented rice substrate was extracted with EtOAc (500 mL × 3 times) three times, and the organic solvent was dried under a vacuum to afford 3.7 g of crude extract. The original extract was fractionated on a silica gel column using petroleum ether-acetone (50:1–1:1) progressively to give ten fractions (fraction 1 to fraction 10). Fraction 3 (120 mg) was purified by semi-preparative HPLC (UV 210nm, 75% MeOH in H_2_O) to obtain compound **1** (20.0 mg, *t*_R_ 19.3 min). Fraction 5 (124 mg) was purified by semi-preparative HPLC (UV 210 nm, 55% MeOH in H_2_O) to yield compound **3** (9.0 mg, *t*_R_ 15.2 min). Fraction 6 (233 mg) was subjected to silica gel using petroleum ether-acetone (6:1) isocratic elution to give five subfractions (fraction 6.1 to fraction 6.5). Fraction 6.2 (90 mg) was then purified by semi-preparative HPLC (UV 210 nm, 55% MeOH in H_2_O) to yield compound **6** (6.8 mg, *t*_R_ 14.9 min). Fraction 6.4 (31 mg) was then purified by semi-preparative HPLC (UV 210 nm, 55% MeOH in H_2_O) to yield compound **5** (11.6 mg, *t*_R_ 14.5 min) and compound **7** (21.7 mg, *t*_R_ 14.3 min). Fraction 8 (86 mg) was purified by semi-preparative HPLC (UV 210 nm, 50% MeOH in H_2_O) to obtain compound **2** (2.6 mg, *t*_R_ 14.9 min) and compound **4** (5.6 mg, *t*_R_ 12.5 min)

*Rhinoclactone A* (**1**) was colorless oil, ${\left[\alpha \right]}_{D}^{20}$ +10.0° (c 0.1, MeOH), UV (MeOH) λ_max_: 292 nm, IR (KBr)*ν*_max_: 3412, 2930, 2852, 1717, 1587, 1464, 1257, 1214, 1108 and 1023 cm^−1^. HR-ESI-MS *m*/*z*: 391.1289 [M + Na]^+^ (calcd for C_19_H_25_ClO_5_Na, 391.1288). ^1^H-(Acetone-*d*_6_, 600 MHz) and ^13^C-(Acetone-*d*_6_, 150 MHz) NMR data; see Table 1.

*Rhinoclactone B* (**2**) was colorless oil, ${\left[\alpha \right]}_{D}^{20}$ −42.0° (c 0.1, MeOH), UV (MeOH) λ_max_: 205 nm, IR (KBr)*ν*_max_: 3459, 2931, 1708, 1588, 1463, 1211, 993 and 835 cm^−1^. HR-ESI-MS *m*/*z*: 407.1238 [M + Na]^+^ (calcd for C_19_H_25_ClO_6_Na, 407.1237). ^1^H-(Acetone-*d*_6_, 600 MHz) and ^13^C-(Acetone-*d*_6_, 150 MHz) NMR data; see Table 1.

*Rhinoclactone C* (**4**) was colorless oil, ${\left[\alpha \right]}_{D}^{20}$ −12.0° (c 0.1, MeOH), UV (MeOH) λ_max_: 270 nm; IR (KBr)*ν*_max_: 3398, 2935, 1714, 1589, 1456, 1257, 1102 and 834 cm^−1^. HR-ESI-MS *m*/*z*: 421.1031 [M + Na]^+^ (calcd for C_19_H_23_ClO_7_Na, 421.1030). ^1^H-(Acetone-*d*_6_, 600 MHz) and ^13^C-(Acetone-*d*_6_, 150 MHz) NMR data; see Table 1.

*Rhinoclactone D* (**5**) was colorless oil, ${\left[\alpha \right]}_{D}^{20}$ −12.0° (c 0.1, MeOH), UV (MeOH) λ_max_: 202 nm, IR (KBr)*ν*_max_: 3389, 2923, 2848, 1718, 1674, 1249 and 1027 cm^−1^. HR-ESI-MS *m*/*z*: 435.1187 [M + Na]^+^ (calcd for C_20_H_25_ClO_7_Na, 435.1187). ^1^H-(Acetone-*d*_6_, 600 MHz) and ^13^C-(Acetone-*d*_6_, 150 MHz) NMR data; see Table 1.

*Rhinoclactone E* (**6**) was colorless oil, ${\left[\alpha \right]}_{D}^{20}$ +36.0° (c 0.1, MeOH), UV (MeOH) λ_max_: 208nm; IR (KBr)*ν*_max_: 3390, 2921, 2850, 1715, 1599, 1457, 1255 and 1100 cm^−1^. HR-ESI-MS *m*/*z*: 435.1185 [M + Na]^+^ (calcd for C_20_H_25_ClO_7_Na, 435.1187). ^1^H-(Acetone-*d*_6_, 600 MHz) and ^13^C-(Acetone-*d*_6_, 150 MHz) NMR data; see Table 1.

### 3.4. X-ray Crystallographic Analysis of ***1***

Upon crystallization from CH_3_OH–H_2_O (10:1) using the vapor diffusion method, colorless crystals were obtained for **1**. A crystal (0.15 mm × 0.14 mm × 0.10 mm) was separated from the sample and the crystal data were obtained on the Agilent GEMINITME instrument (CrysAlisPro software, Version 1.171.35.11, Palo Alto, CA, USA) with enhanced Cu Kα radiation (*λ* = 1.54184 Å). The structure solution and refinement were performed with the SHELXL-97 by full-matrix least-square techniques. Crystal data was C_19_H_25_ClO_5_, M = 368.84, orthorhombic, space group *P*2_1_2_1_2_1_ with unit cell dimensions *a* = 7.75828 (14) Å, *b* = 15.2428(3) Å, *c* = 15.3442(3), *V* = 1814.57(6) Å^3^, *Z* = 4, T = 107.3 K, *ρ_calcd_* = 1.350 mg mm^−3^, *μ* (Cu Kα) = 2.091 mm^−1^, *F* (000) = 784. A total of 6157 reflections were collected in the range 11.53° < *θ* < 142.27° with 3435 independent reflections [*R*(int) = 0.0245]. The final *R* indexes [*I* > 2σ(*I*)] were *R*_1_ = 0.0325, *wR*_2_ = 0.0823, and *R* indexes (all data) were *R*_1_ = 0.0338, *wR*_2_ = 0.0834. The flack parameter was −0.006(10). The crystallographic data for the structure of **1** was deposited in the Cambridge Crystallographic Data Centre [deposition number: CCDC 1580280].

### 3.5. Cytotoxic Evaluation (MTT Assay)

The cytotoxic activities of isolated **1**–**7** were evaluated against HCT116, MCF7 and HeLa cancer cell lines on the basis of the MTT (3-(4,5-dimethylthiazol-2-yl)-2,5-diphenyltetrazolium bromide) colorimetric approach with the *cis*-platinum as the positive control. The procedure of cytotoxic evaluation described was as in Reference [19].

## 4. Conclusions

Seven RALs including five new analog rhinoclactones A–E (**1**, **2**, **4**–**6**) were isolated from the endophytic fungus *R. similis*, which diversified the structural types of RALs. Our results implied that endophytic fungi from desert plants will be a potential resource for finding bioactive secondary metabolites, which will benefit the drug discovery and could elucidate the, potentially, ecological roles between endophytic fungi and their plant hosts.

## Figures and Tables

**Table 1:** NMR spectroscopic data for compounds **1**, **2, 4**–**6** in Acetone-*d*_6_ (*δ* in ppm, *J* in Hz).

No.	1		2		4		5		6	
	*δ*_H_^a^	*δ*_C_^b^	*δ*_H_^a^	*δ*_C_^b^	*δ*_H_^a^	*δ*_C_^b^	*δ*_H_^a^	*δ*_C_^b^	*δ*_H_^a^	*δ*_C_^b^
1	1.27, d (7.2)	19.6	1.28, d (7.8)	19.6	1.28, d (7.8)	19.5	1.27, d (7.2)	20.6	1.29, d (7.2)	19.5
2	5.17, m	70.4	5.20, m	72.0	5.12, m	70.9	4.90, m	71.4	5.16, m	70.9
3	1.60, m	34.7	1.48, m	30.8	2.16, m	27.9	1.50, m	29.5	2.21, m	27.9
	1.60, m		1.28, m		1.60, m		1.50, m		1.58, m	
4	1.33, m	22.1	1.51, m	31.6	2.62, m	37.3	2.64, ddd (18.5, 10.0, 4.0)	35.2	2.66, m	35.4
	1.33, m		1.28, m		2.37, m		2.16, ddd (18.5, 5.0, 5.0)		2.38, m	
5	1.36, m	25.7	3.45, m	70.3		209.7		209.6		209.6
	1.36, m									
6	1.34, m	24.9	1.64, m	34.6	2.64, m	35.2	2.43, ddd (15.0, 7.5, 3.5)	40.6	2.55, m	36.7
	1.34, m		1.29, m		2.64, m		2.24, ddd (15.0, 11.5, 3.5)		2.34, m	
7	1.70, m	22.7	1.40, m	22.4	1.87, m	30.2	2.09, m	21.3	1.89, m	26.6
	1.50, m		1.40, m		1.87, m		1.76, m		1.89, m	
8	1.70, m	25.9	1.41, m	22.8	3.82, m	65.6	3.56, m	79.1	3.37, m	75.3
	1.52, m		1.17, m							
8-OMe							3.27 s	57.1	3.27 s	55.5
9	2.71, ddd (14.5, 9.5, 3.0)	40.5	2.79, ddd (17.0, 7.5, 3.0)	39.5	2.63, dd (14.0, 3.5)	48.8	3.00, dd (14.5, 8.0)	45.5	2.68, dd (14.5, 3.5)	45.2
	2.30, ddd (14.5, 9.0, 3.0)		2.46, ddd (17.0, 10.5, 3.5)		2.33, dd (14.0, 9.5)		2.52, dd (14.5, 1.5)		2.21, dd (14.5, 9.0)	
10		204.4		204.1		203.9		205.0		203.4
11	4.13, d (18.5)	44.1	4.03, d (18.5)	44.5	4.10, d (18.0)	45.3	4.07, d (18.5)	46.8	4.12, d (17.5)	44.9
	3.97, d (18.5)		3.97, d (18.5)		3.89, d (18.0)		4.01, d (18.5)		3.89, d (17.5)	
12		132.6		132.3		132.4		133.2		132.4
13		113.7		113.5		113.5		114.8		113.4
14		154.9		154.6		155.3		156.8		155.2
15	6.68, s	99.3	6.68, s	99.2	6.71, s	99.5	6.67, s	100.5	6.69, s	99.5
16		156.4		156.1		156.6		157.3		156.7
17		117.9		118.6		117.8		118.6		117.9
18		167.0		166.7		167.0		168.2		167.0
16-OMe	3.77, s	55.5	3.79, s	55.6	3.78, s	55.6	3.74, s	56.5	3.78, s	55.6

^a^ Recorded at 600 MHz. ^b^ Recorded at 150 MHz.

## Supplementary Materials

The following are available online.
