# Supplementary material for: Rhinoclactones A-E, Resorcylic Acid Analogs from Desert Plant Endophytic Fungus Rhinocladiella similis
Source: Molecules. 2019 Apr 10;24(7):1405. doi: 10.3390/molecules24071405 (PMC6480478; doi:10.3390/molecules24071405)
Supplement: Supplementary file 1 [file molecules-24-01405-s001.pdf]

# Supporting Information

## Rhinoclactones A-E, Resorcylic Acid Analogs from Desert Plant Endophytic Fungus *Rhinocladiella similis*

Luying Li[1, †], Xiaoyan Zhang[1, †], Xiangmei Tan[1], Bingda Sun[2], Bin Wu[1, *], Meng Yu[1], Tao Zhang[1], Yonggang Zhang[3, *] Gang Ding[1, *]

*Correspondence and requests for materials should be addressed to Prof., Dr. Gang Ding or Prof., Dr. Yonggang Zhang or Prof., Dr. Bin Wu. (Email: dgfyhchina@163.com or zhangygcq@163.com or bwu@implad.ac.cn, Tel: +86 010 57833290; fax: +86 010 57833290)

## Contents of Supplementary Information


| | Contents |
|---|---|
| Tab.S1 | $^{1}H$ NMR data (600 MHz, Acetone-$d_6$) of the diagnostic protons from the (*S*)- and (*R*)- OMTPA esters derivatives of compound **2** |
| Tab.S2 | $^{1}H$ NMR data (600 MHz, Acetone-$d_6$) of the diagnostic protons from the (*S*)- and (*R*)- OMTPA esters derivatives of compound **4** |
| Fig. S1.1 | $^{1}H$ NMR spectrum (600 MHz) of compound (**1**) in Acetone-$d_6$ |
| Fig. S1.2 | $^{13}C$ NMR spectrum (150 MHz) of compound (**1**) in Acetone-$d_6$ |
| Fig. S1.3 | HSQC spectrum (600 MHz) of compound (**1**) in Acetone-$d_6$ |
| Fig. S1.4 | HMBC spectrum (600 MHz) of compound (**1**) in Acetone-$d_6$ |
| Fig. S1.5 | $^{1}H$-$^{1}H$ COSY spectrum (600 MHz) of compound(**1**) in Acetone-$d_6$ |
| Fig. S1.6 | UV spectrum of compound (**1**) |
| Fig. S1.7 | IR spectrum of compound (**1**) |
| Fig. S1.8 | HR-ESI-MS spectrum of compound (**1**) |
| Fig. S2.1 | $^{1}H$ NMR spectrum (600 MHz) of compound (**2**) in Acetone-$d_6$ |
| Fig. S2.2 | $^{13}C$ NMR spectrum (150 MHz) of compound (**2**) in Acetone-$d_6$ |
| Fig. S2.3 | HSQC spectrum (600 MHz) of compound (**2**) in Acetone-$d_6$ |
| Fig. S2.4 | HMBC spectrum (600 MHz) of compound (**2**) in Acetone-$d_6$ |
| Fig. S2.5 | $^{1}H$-$^{1}H$ COSY spectrum (600 MHz) of compound(**2**) in Acetone-$d_6$ |
| Fig. S2.6 | UV spectrum of compound (**2**) |
| Fig. S2.7 | IR spectrum of compound (**2**) |
| Fig. S2.8 | HR-ESI-MS spectrum of compound (**2**) |
| Fig. S3.1 | $^{1}H$ NMR spectrum (600 MHz) of compound (**4**) in Acetone-$d_6$ |
| Fig. S3.2 | $^{13}C$ NMR spectrum (150 MHz) of compound (**4**) in Acetone-$d_6$ |
| Fig. S3.3 | HSQC spectrum (600 MHz) of compound (**4**) in Acetone-$d_6$ |
| Fig. S3.4 | HMBC spectrum (600 MHz) of compound (**4**) in Acetone-$d_6$ |
| Fig. S3.5 | UV spectrum of compound (**4**) |
| Fig. S3.6 | IR spectrum of compound (**4**) |
| Fig. S3.7 | HR-ESI-MS spectrum of compound (**4**) |
| Fig. S4.1 | $^{1}H$ NMR spectrum (600 MHz) of compound (**5**) in Acetone-$d_6$ |
| Fig. S4.2 | $^{13}C$ NMR spectrum (150 MHz) of compound (**5**) in Acetone-$d_6$ |
| Fig. S4.3 | HSQC spectrum (600 MHz) of compound (**5**) in Acetone-$d_6$ |
| Fig. S4.4 | HMBC spectrum (600 MHz) of compound (**5**) in Acetone-$d_6$ |
| Fig. S4.5 | $^{1}H$-$^{1}H$ COSY spectrum (600 MHz) of compound (**5**) in Acetone-$d_6$ |
| Fig. S4.6 | UV spectrum of compound (**5**) |
| Fig. S4.7 | IR spectrum of compound (**5**) |
| Fig. S4.8 | HR-ESI-MS spectrum of compound (**5**) |
| Fig. S5.1 | $^{1}H$ NMR spectrum (600 MHz) of compound (**6**) in Acetone-$d_6$ |
| Fig. S5.2 | $^{13}C$ NMR spectrum (150 MHz) of compound (**6**) in Acetone-$d_6$ |
| Fig. S5.3 | HSQC spectrum (600 MHz) of compound (**6**) in Acetone-$d_6$ |
| Fig. S5.4 | HMBC spectrum (600 MHz) of compound (**6**) in Acetone-$d_6$ |
| Fig. S5.5 | $^{1}H$-$^{1}H$ COSY spectrum (600 MHz) of compound (**6**) in Acetone-$d_6$ |

| | |
|---|---|
| Fig. S5.6 | UV spectrum of compound (**6**) |
| Fig. S5.7 | IR spectrum of compound (**6**) |
| Fig. S5.8 | HR-ESI-MS spectrum of compound (**6**) |
| Fig. S6.1 | $^{1}$H NMR spectrum (600 MHz) of **2a** in Acetone-$d_6$ |
| Fig. S6.2 | $^{1}$H NMR spectrum (600 MHz) of **2b** in Acetone-$d_6$ |
| Fig. S6.3 | $^{1}$H-$^{1}$H COSY spectrum (600 MHz) of **2a** in Acetone-$d_6$ |
| Fig. S6.4 | $^{1}$H-$^{1}$H COSY spectrum (600 MHz) of **2b** in Acetone-$d_6$ |
| Fig. S7.1 | $^{1}$H NMR spectrum (600 MHz) of **4a** in Acetone-$d_6$ |
| Fig. S7.2 | $^{1}$H NMR spectrum (600 MHz) of **4b** in Acetone-$d_6$ |
| Fig. S7.3 | $^{1}$H-$^{1}$H COSY spectrum (600 MHz) of **4a** in Acetone-$d_6$ |
| Fig. S7.4 | $^{1}$H-$^{1}$H COSY spectrum (600 MHz) of **4b** in Acetone-$d_6$ |
| Fig. S8.1 | The possible biosynthesis of **1**-**7**. |

**TableS1.**$^{1}$H NMR data (600MHz, Acetone-$d_6$) of the diagnostic protons from the (*S*)- and (*R*)-OMTPA esters derivatives of compound **2**.

| Pos | **2** | | | |
|---|---|---|---|---|
| | $\delta_H$ | $\delta_S$ | $\delta_R$ | $\Delta\delta_H (\delta_S-\delta_R)$ |
| 1 | 1.28 | 1.216 | 1.308 | -0.092 |
| 2 | 5.20 | 5.139 | 5.259 | -0.120 |
| 3 | 1.28<br>1.48 | 1.363<br>1.641 | 1.600<br>1.914 | -0.237<br>-0.273 |
| 5 | 3.45 | 4.962 | 4.965 | -0.003 |
| 9 | 2.79<br>2.46 | 2.890<br>2.549 | 2.856<br>2.507 | +0.034<br>+0.042 |

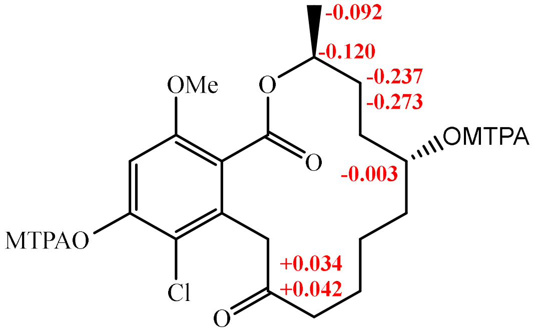


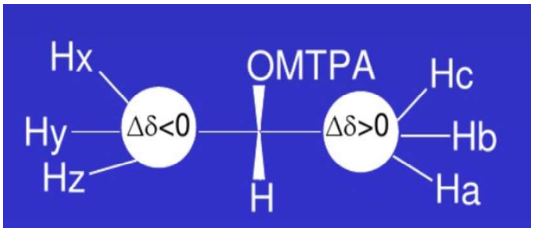

**TableS2.**$^{1}$H NMR data (600MHz, Acetone-$d_6$) of the diagnostic protons from the (*S*)- and (*R*)-OMTPA esters derivatives of compound **4**.

| Pos | 4 | | | |
|---|---|---|---|---|
| | $\delta_H$ | $\delta_S$ | $\delta_R$ | $\Delta\delta_H$ ($\delta_S$-$\delta_R$) |
| 3 | 2.16<br>1.60 | 2.250<br>1.712 | 2.249<br>1.703 | +0.001<br>+0.009 |
| 4 | 2.62<br>2.37 | 2.702<br>2.517 | 2.666<br>2.486 | +0.036<br>+0.031 |
| 6 | 2.64<br>2.64 | 2.645<br>2.443 | 2.443<br>2.332 | +0.202<br>+0.111 |
| 7 | 1.87<br>1.87 | 2.195<br>2.056 | 2.125<br>1.969 | +0.070<br>+0.087 |
| 9 | 2.63<br>2.33 | 2.581<br>2.467 | 2.663<br>2.575 | -0.082<br>-0.108 |
| 11 | 4.10<br>3.89 | 4.067<br>3.905 | 4.082<br>3.933 | -0.015<br>-0.028 |

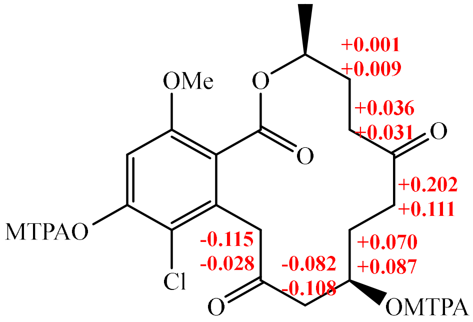


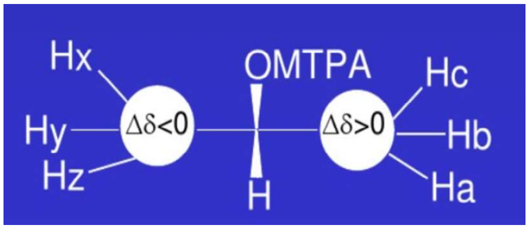

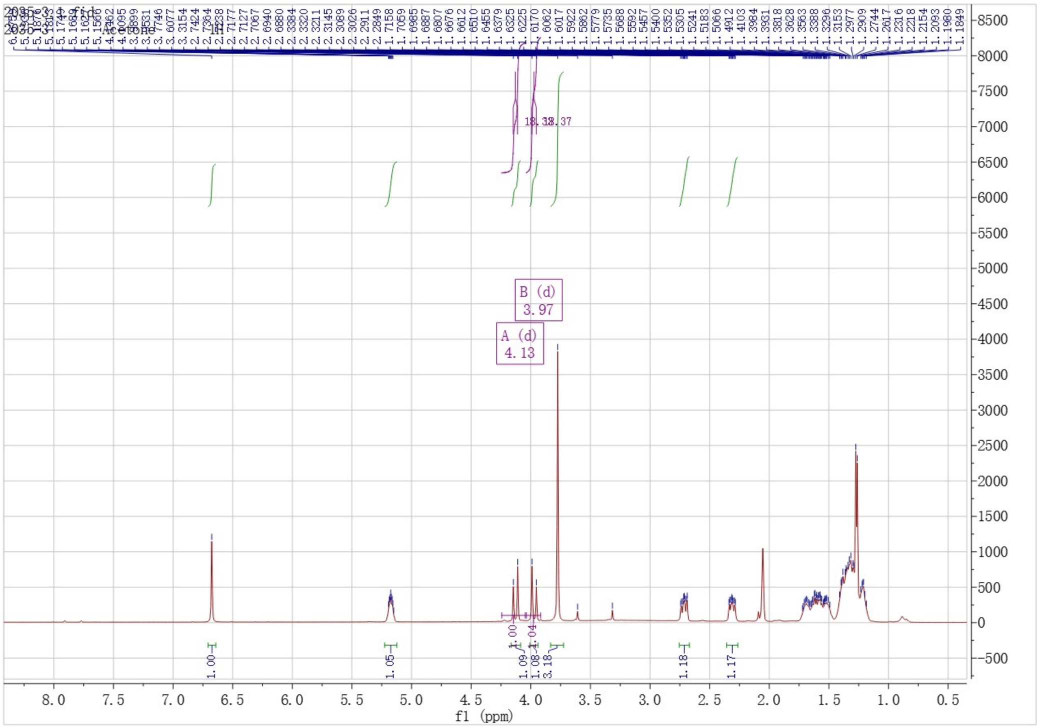


Fig. S1.1 $^{1}$H NMR spectrum of compound (**1**)

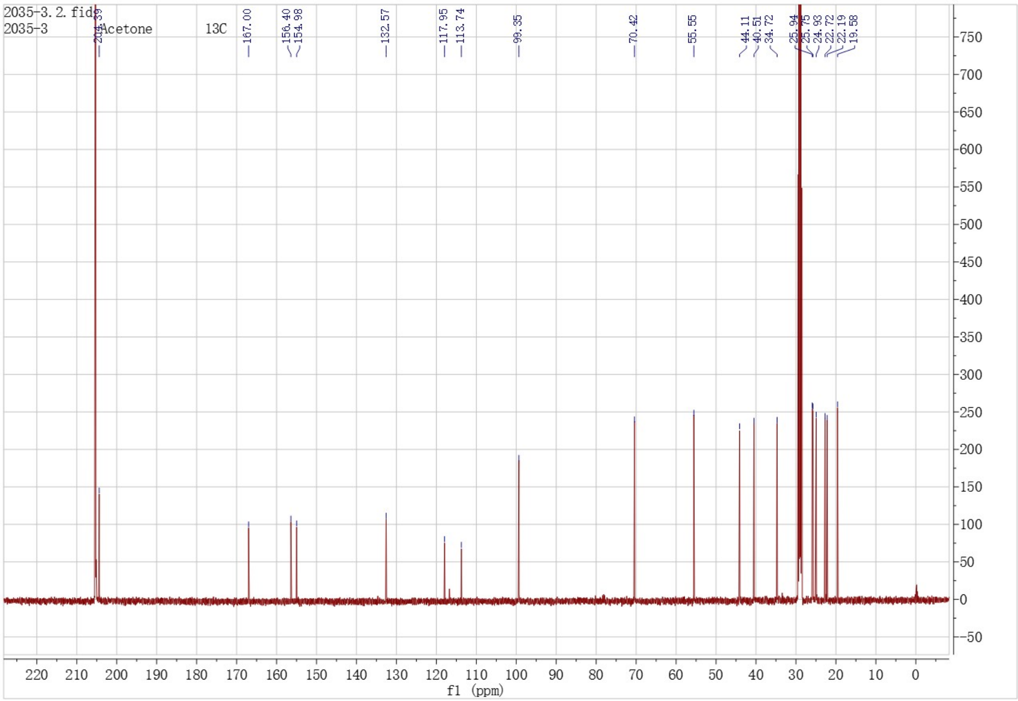


Fig. S1.2 $^{13}$C NMR spectrum of compound (**1**)

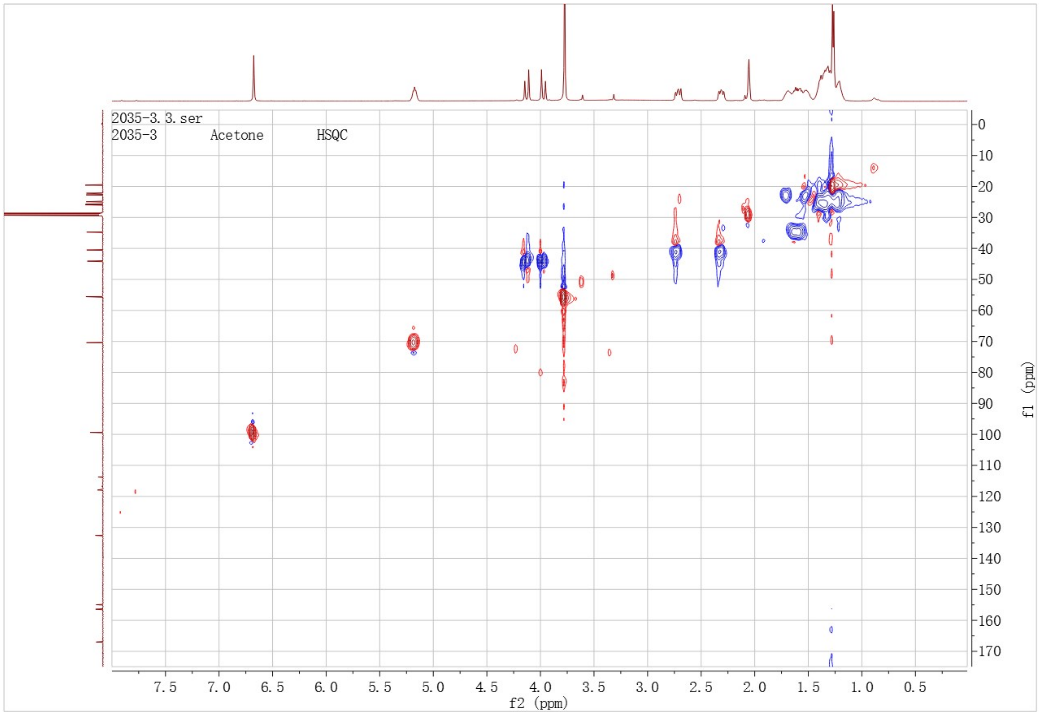


Fig. S1.3 HSQC spectrum of compound (**1**)

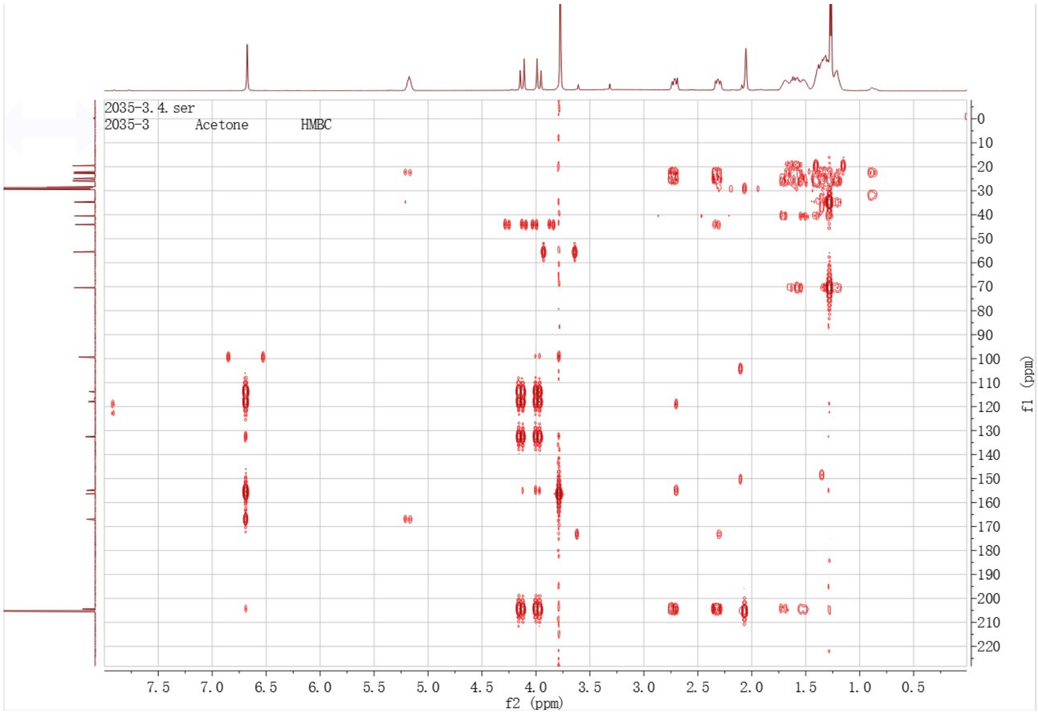


Fig. S1.4 HMBC spectrum of compound (**1**)

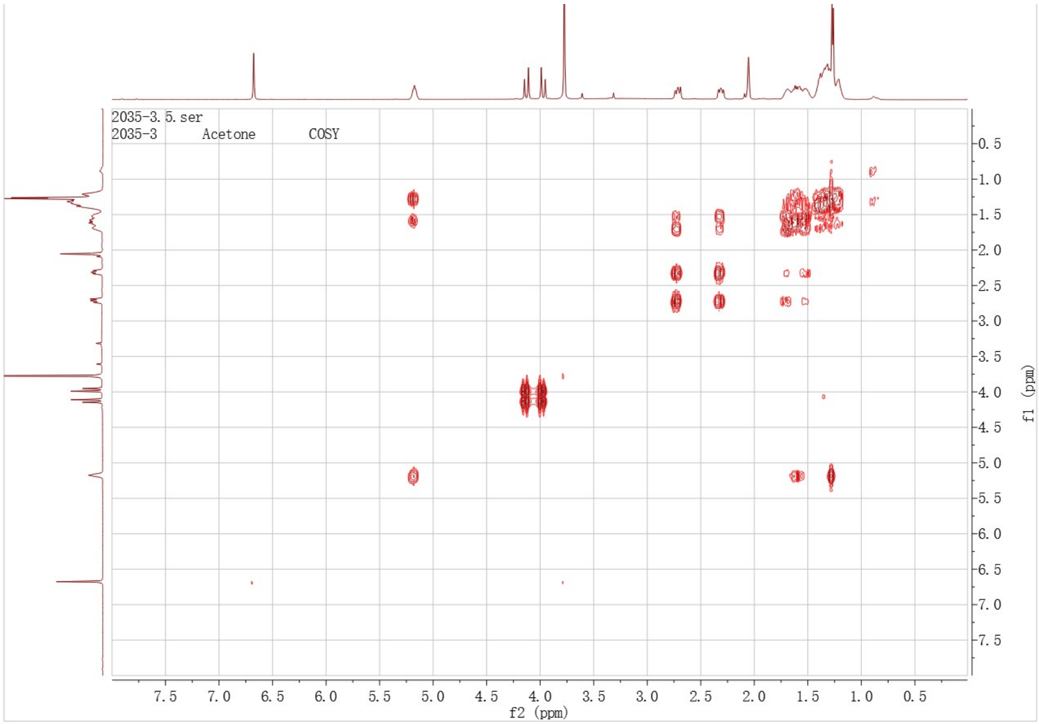


Fig. S1.5 $^{1}H$- $^{1}H$ COSY spectrum of compound (**1**)

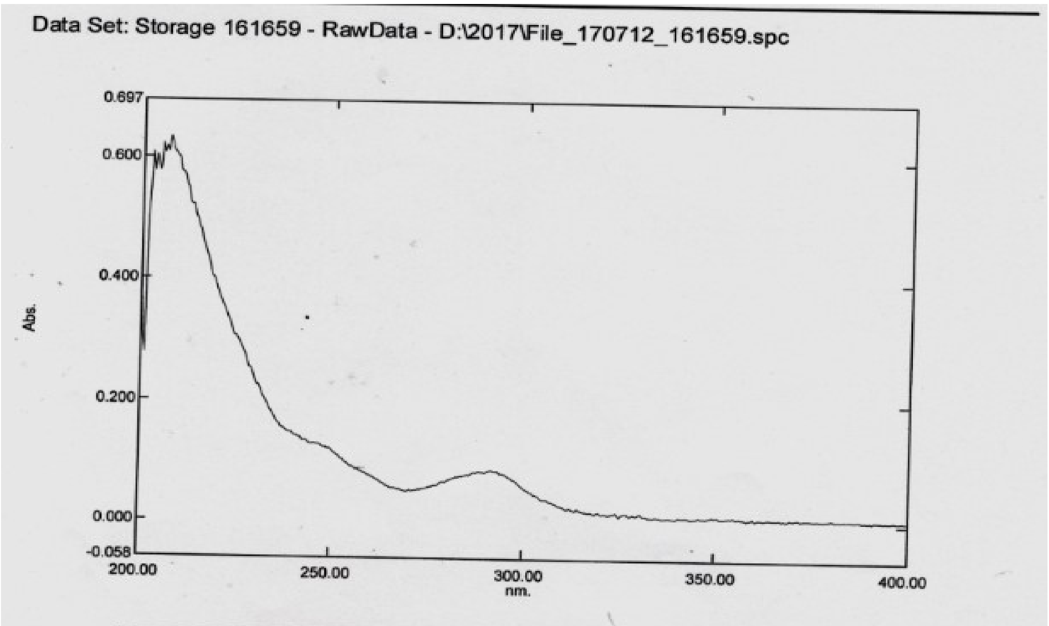


| No. | P/V | Wavelength | Abs. | Description |
|---|---|---|---|---|
| 1 | ● | 355.00 | 0.011 | |
| 2 | ● | 291.50 | 0.085 | |

Fig. S1.6 UV spectrum of compound (**1**)

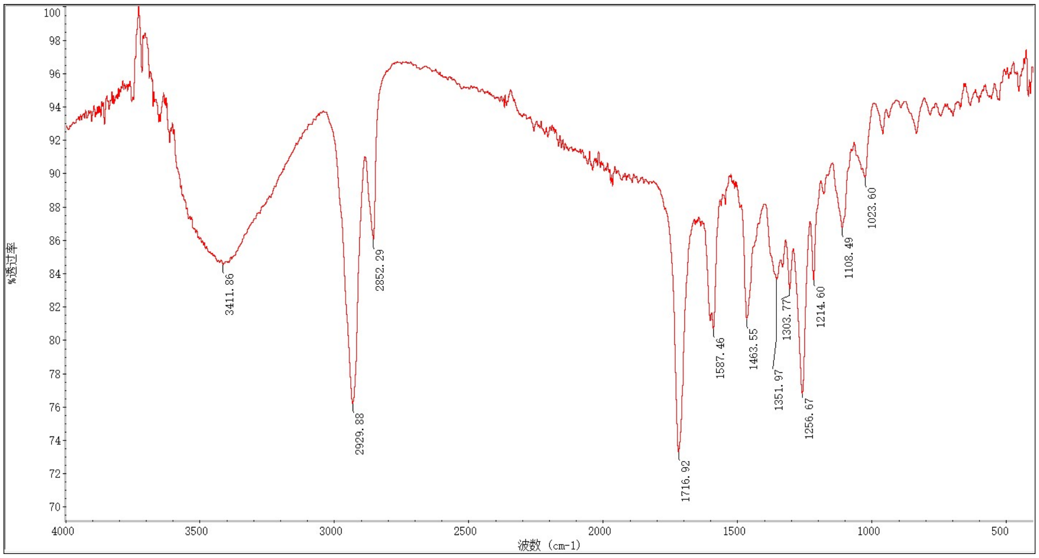


Fig. S1.7 IR spectrum of compound (**1**)

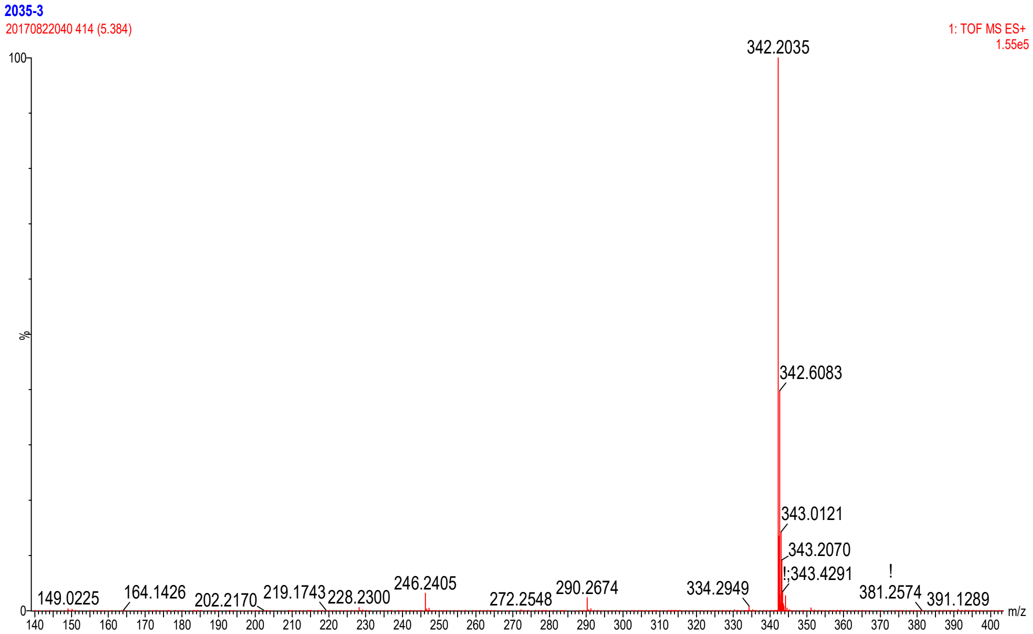


Fig. S1.8 HR-ESI-MS spectrum of compound (**1**)

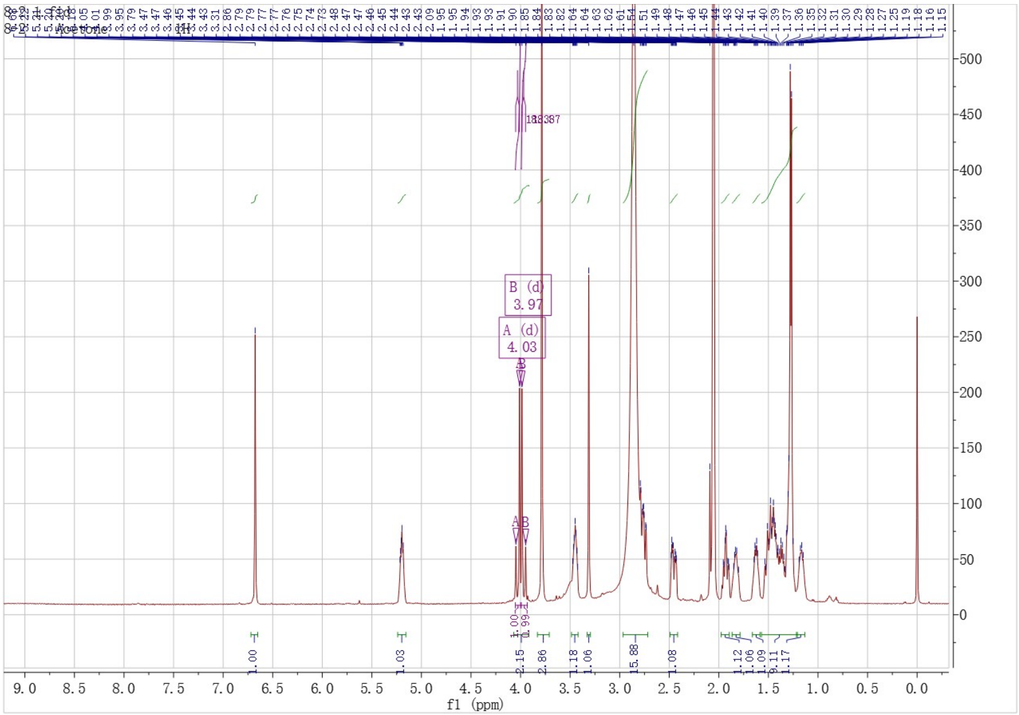

Fig. S2.1 $^{1}$H-NMR spectrum of compound (**2**)

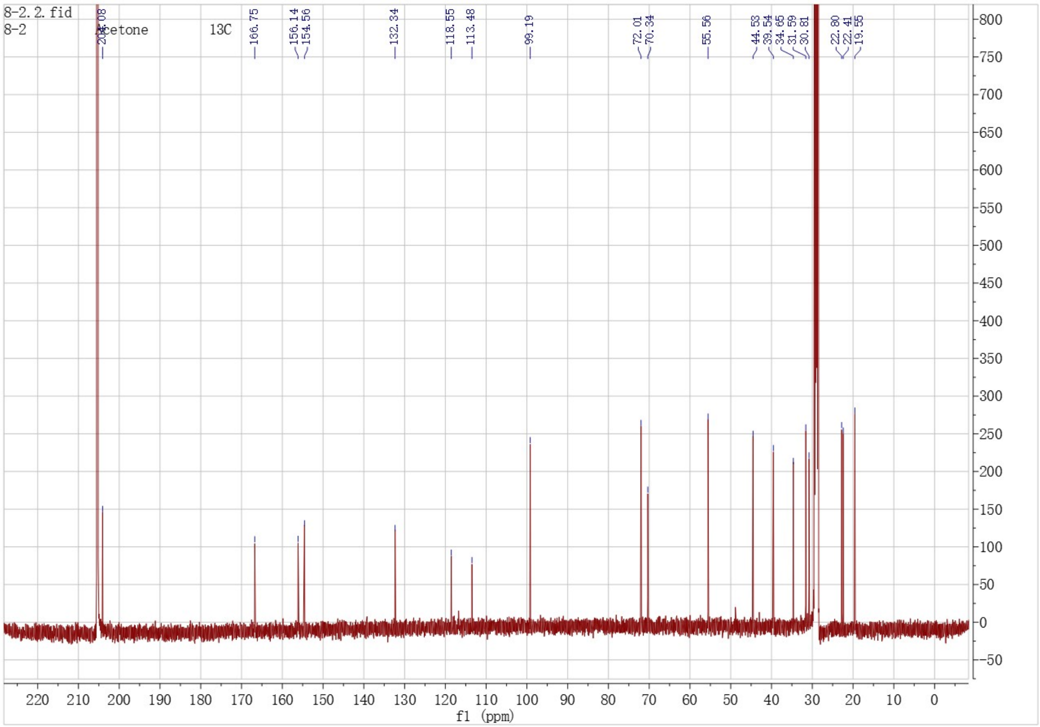

Fig. S2.2 $^{13}$C-NMR spectrum of compound (**2**)

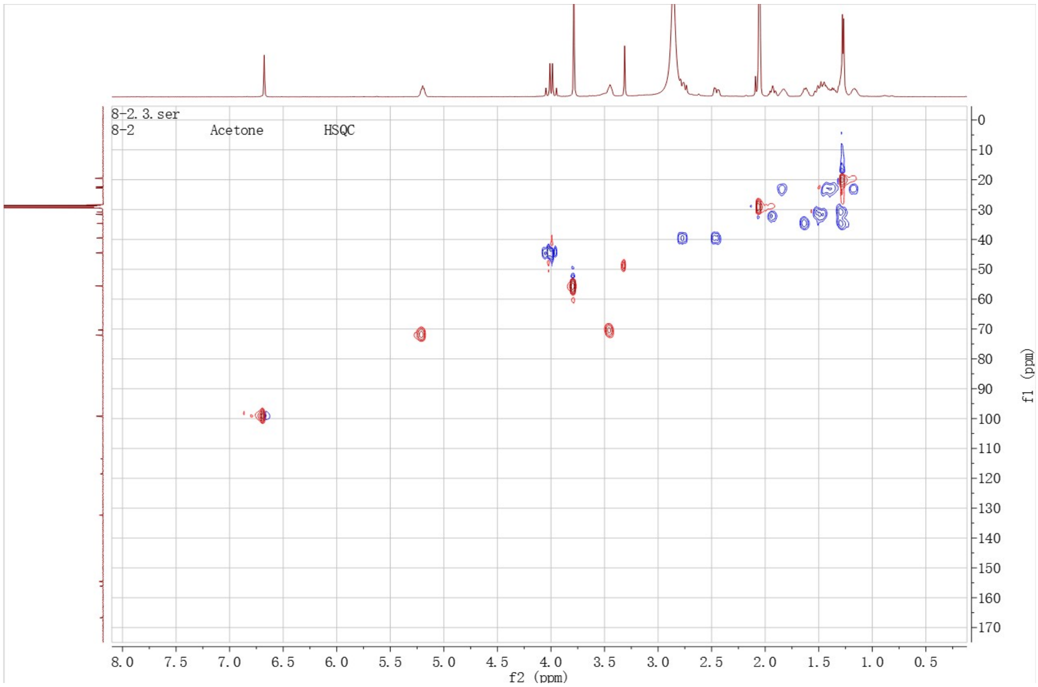


Fig. S2.3 HSQC spectrum of compound (**2**)

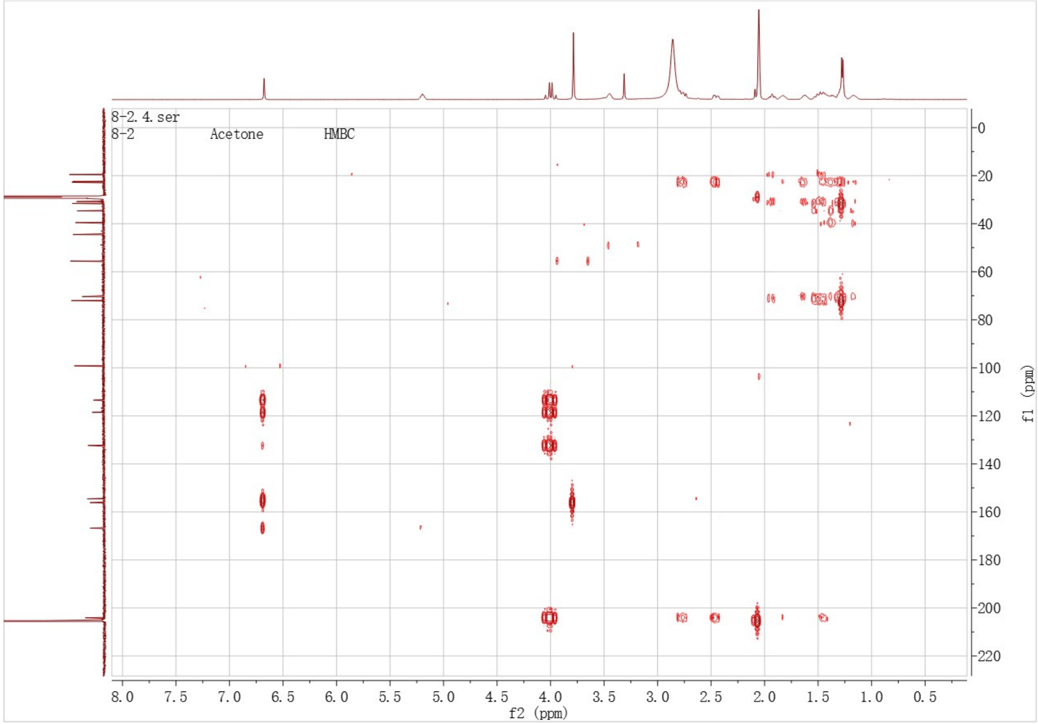


Fig. S2.4 HMBC spectrum of compound (**2**)

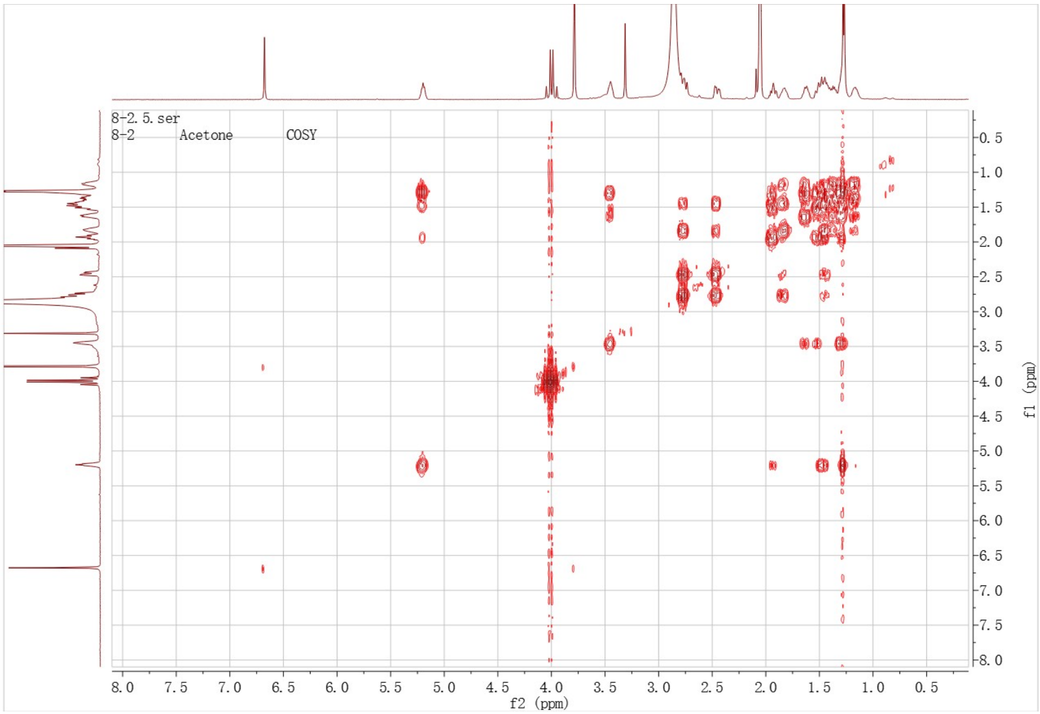


Fig. S2.5 $^{1}$H-$^{1}$HCOSY spectrum of compound (**2**)

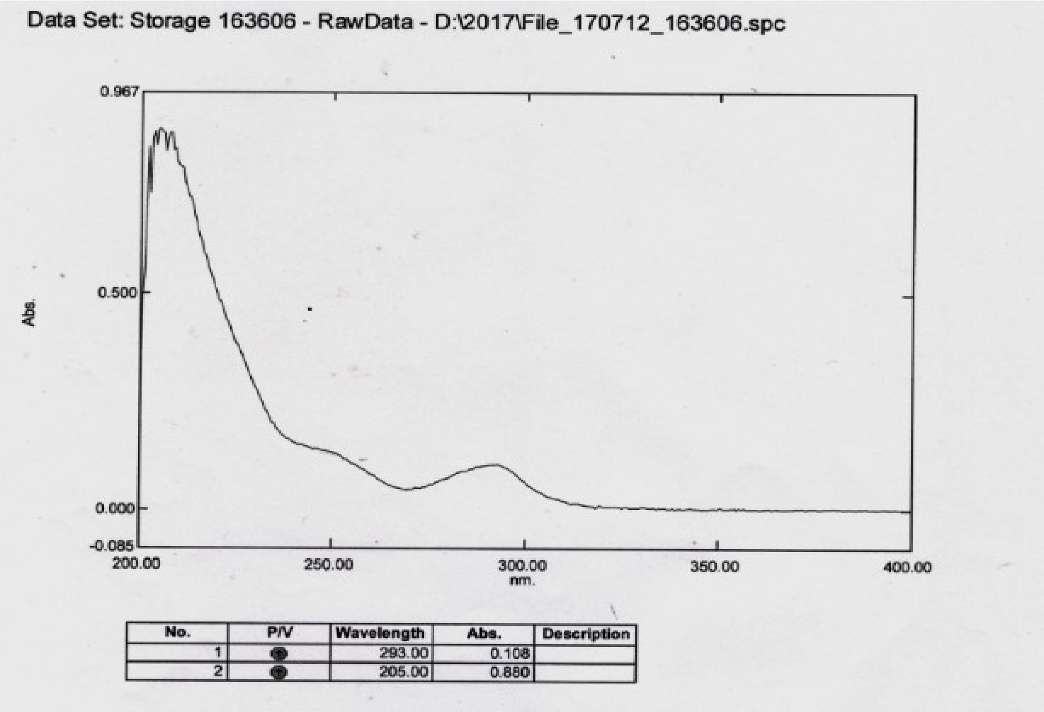


| No. | P/V | Wavelength | Abs. | Description |
|---|---|---|---|---|
| 1 | ⊕ | 293.00 | 0.108 | |
| 2 | ⊕ | 205.00 | 0.880 | |

Fig. S2.6 UV spectrum of compound (**2**)

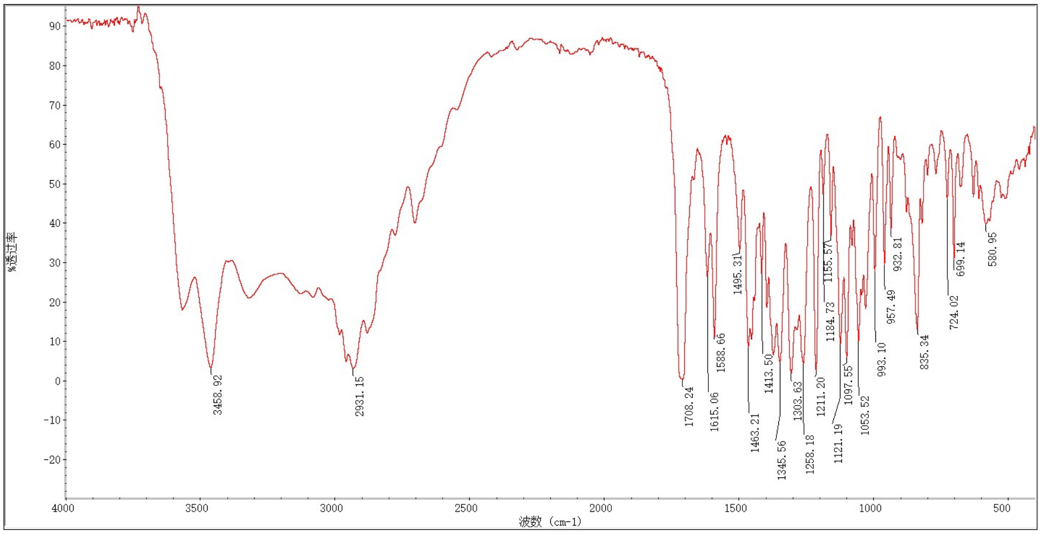


Fig. S2.7 IR spectrum of compound (**2**)

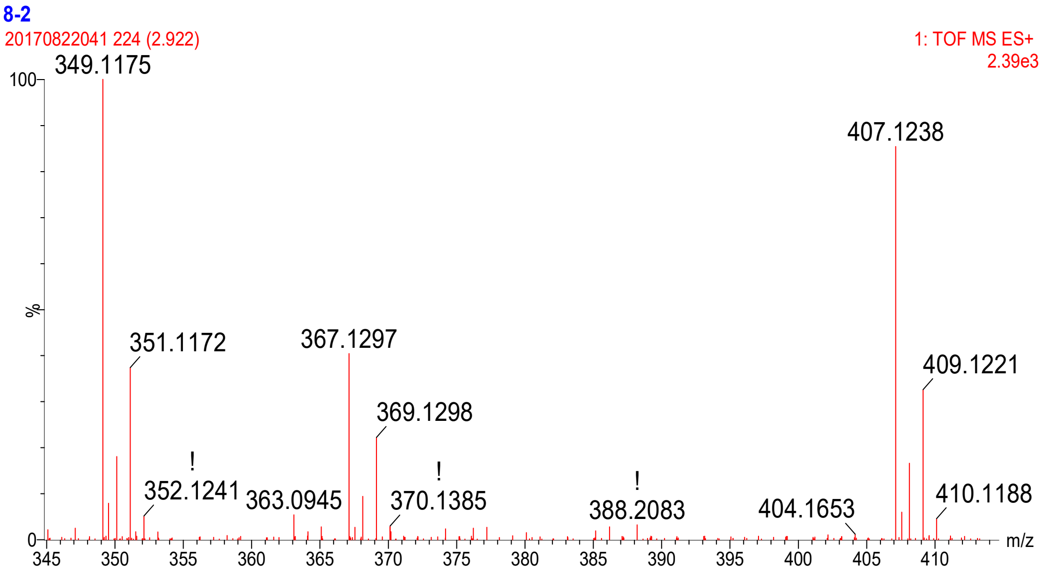


Fig. S2.8 HR-ESI-MS spectrum of compound (**2**)

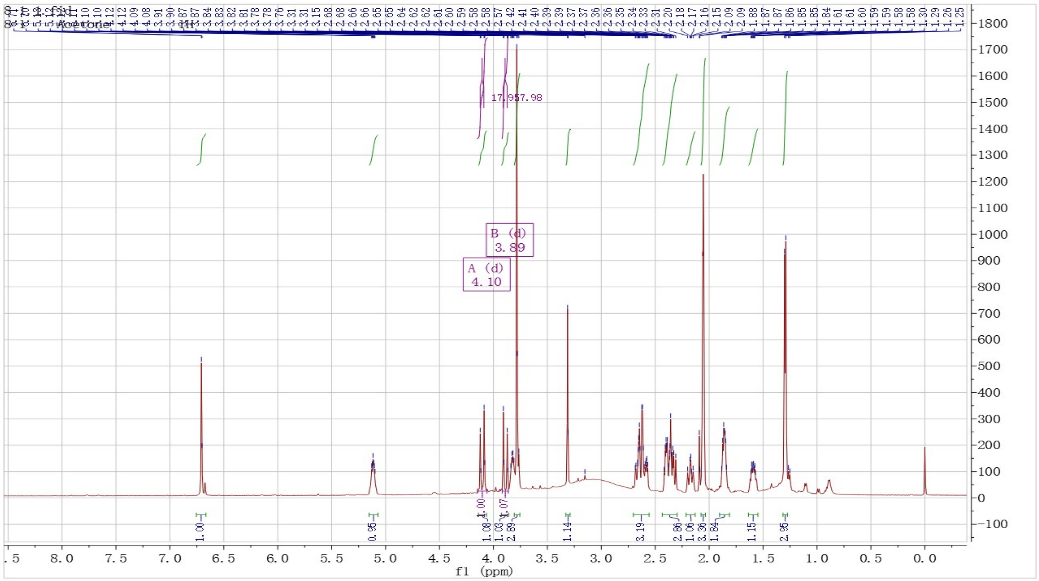

Fig. S3.1 $^{1}H$-NMR spectrum of compound (**4**)

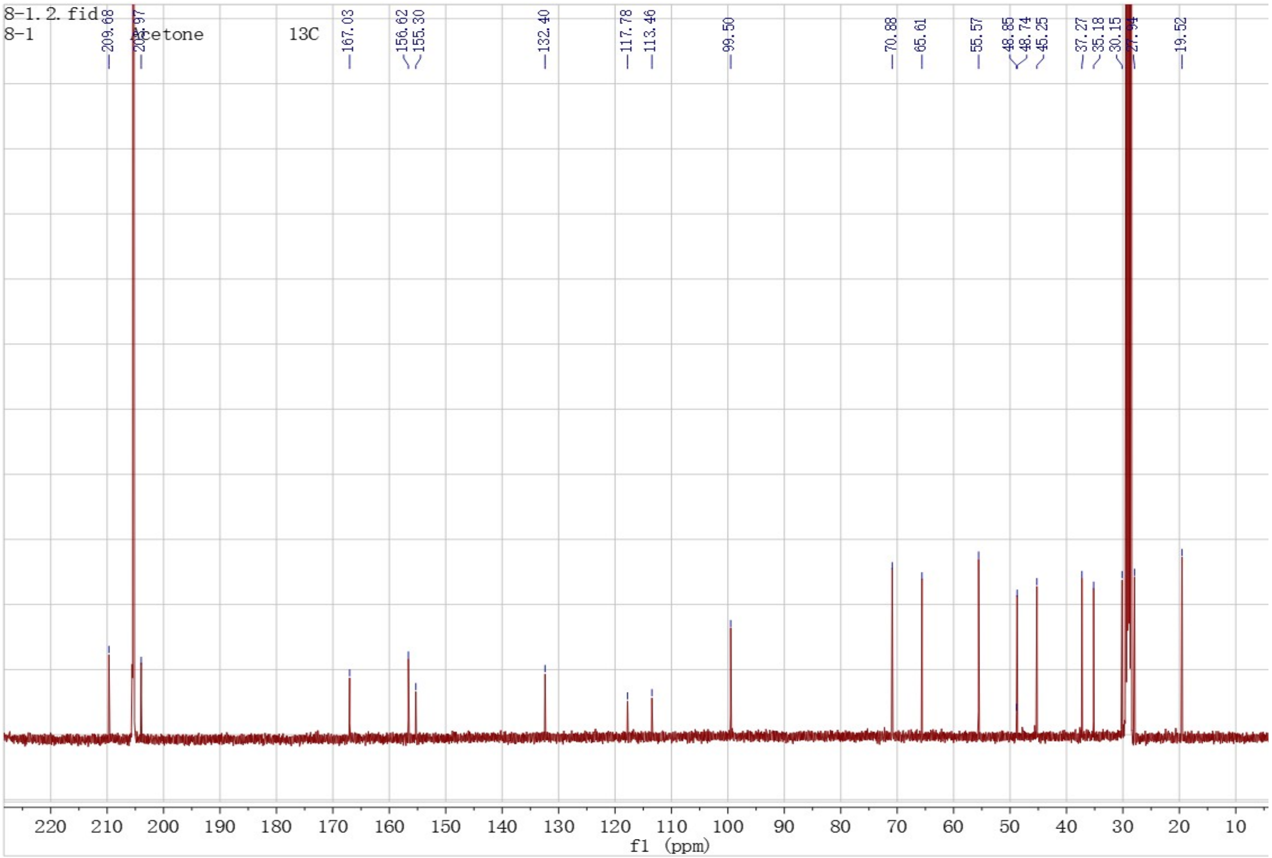


Fig. S3.2 $^{13}C$-NMR spectrum of compound (**4**)

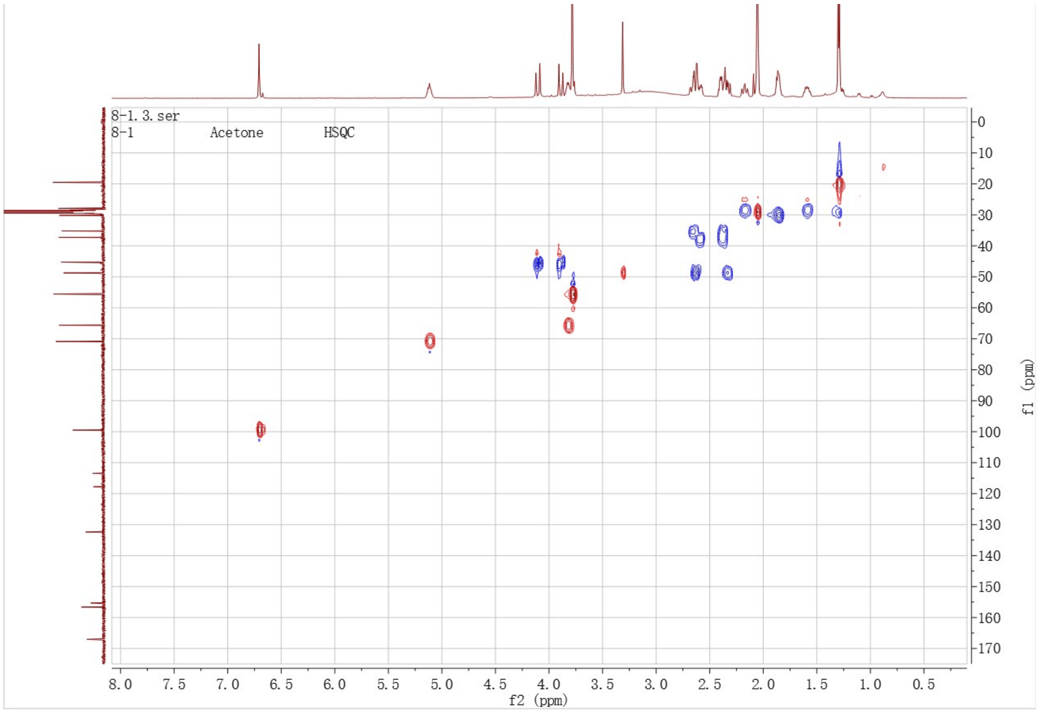


Fig. S3.3 HSQC spectrum of compound (**4**)

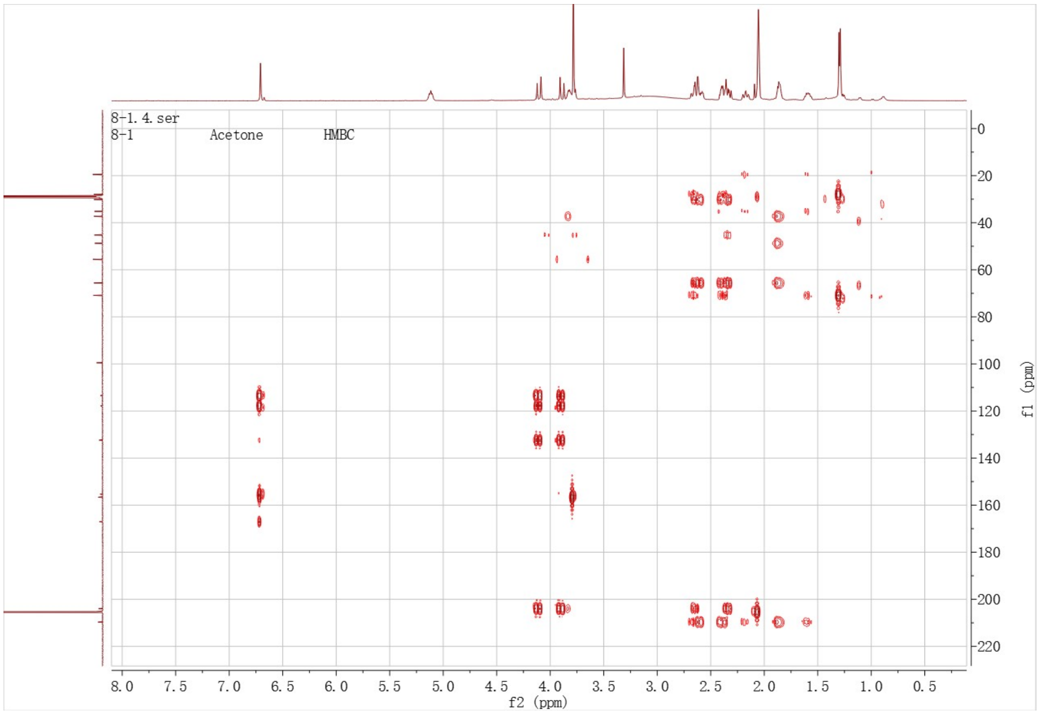

Fig. S3.4 HMBC spectrum of compound (**4**)

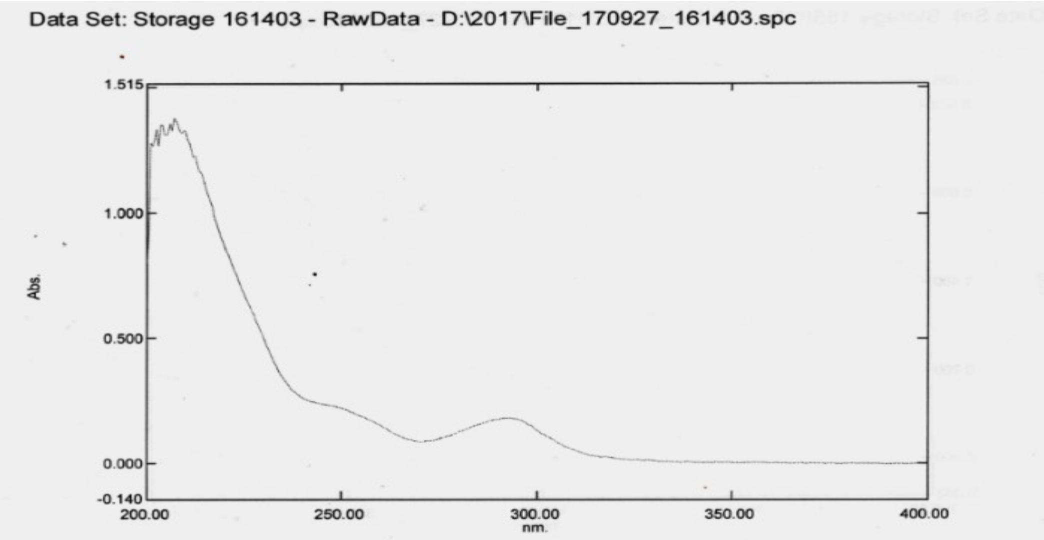


| No. | P/V | Wavelength | Abs. | Description |
|---|---|---|---|---|
| 1 | ↑ | 361.50 | 0.004 | |
| 2 | ↑ | 339.00 | 0.009 | |
| 3 | ↑ | 292.00 | 0.181 | |
| 4 | ↓ | 359.50 | 0.001 | |
| 5 | ↓ | 337.00 | 0.004 | |
| 6 | ↓ | 270.50 | 0.087 | |

Fig. S3.5 UV spectrum of compound (**4**)

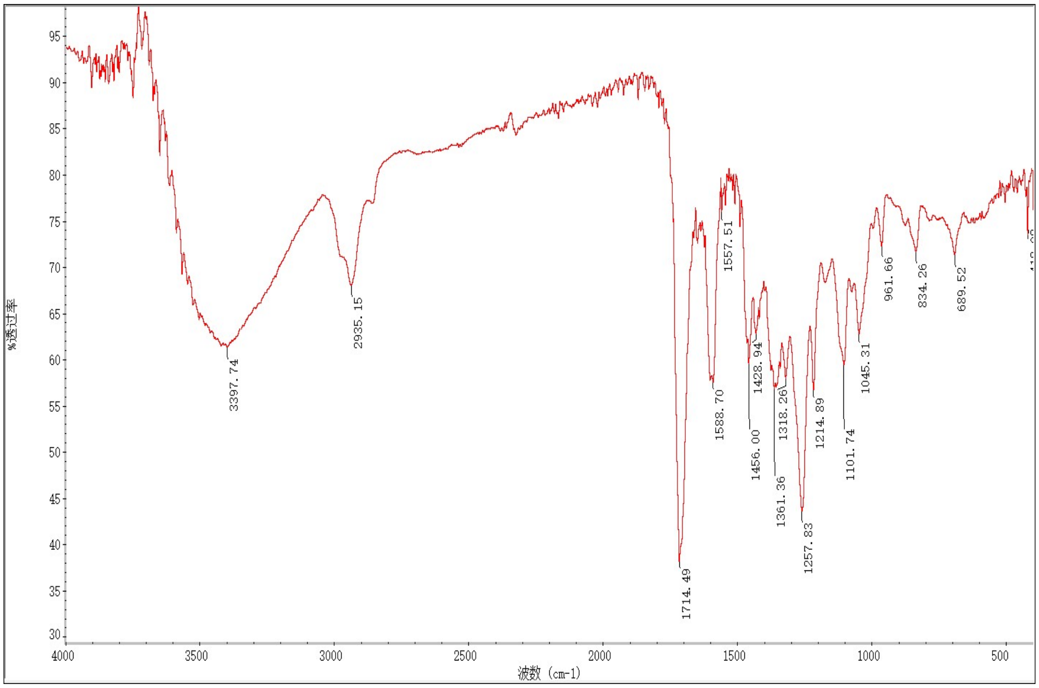

Fig. S3.6 IR spectrum of compound (**4**)

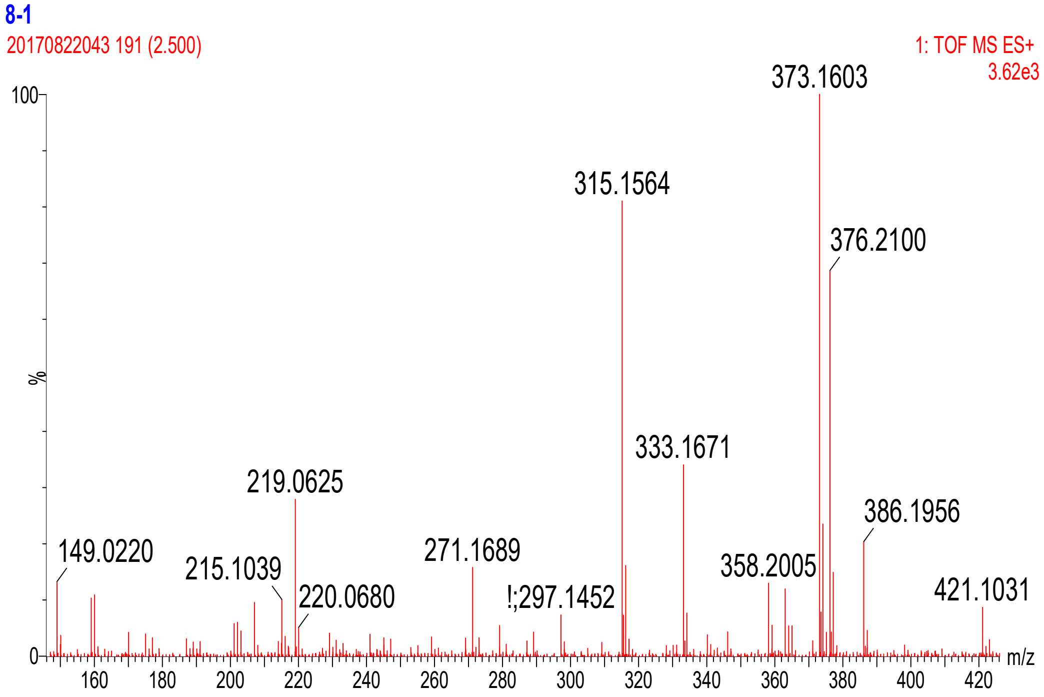


Fig. S3.7 HR-ESI-MS spectrum of compound (**4**)

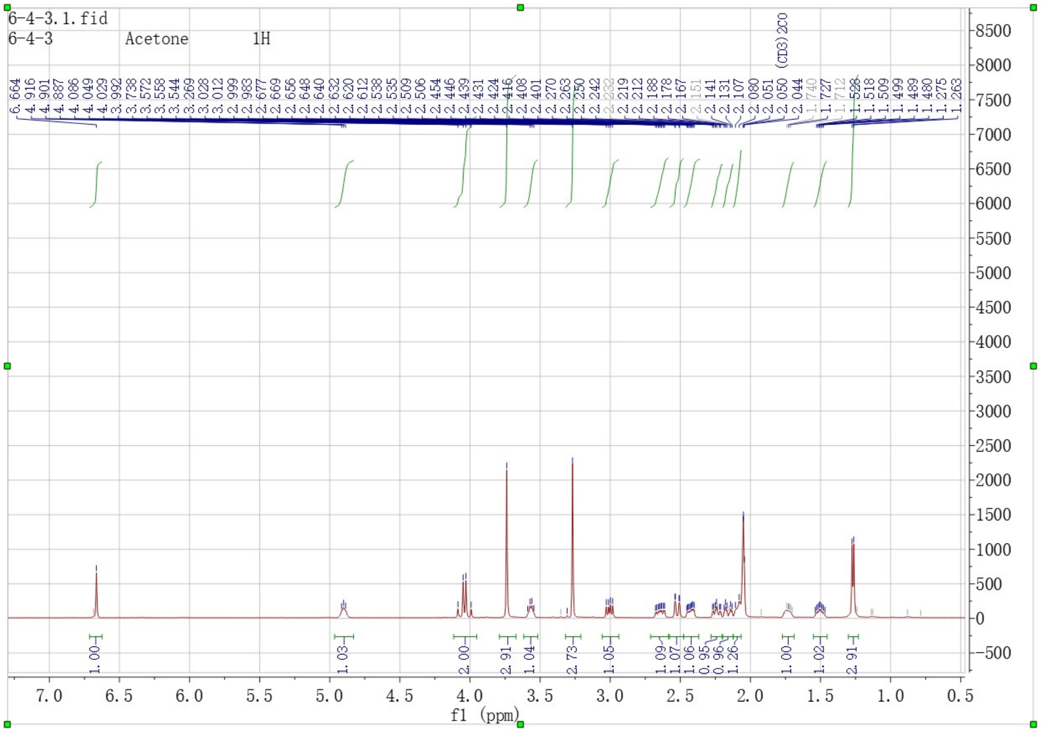

Fig. S4.1 ¹H-NMR spectrum of compound (**5**)

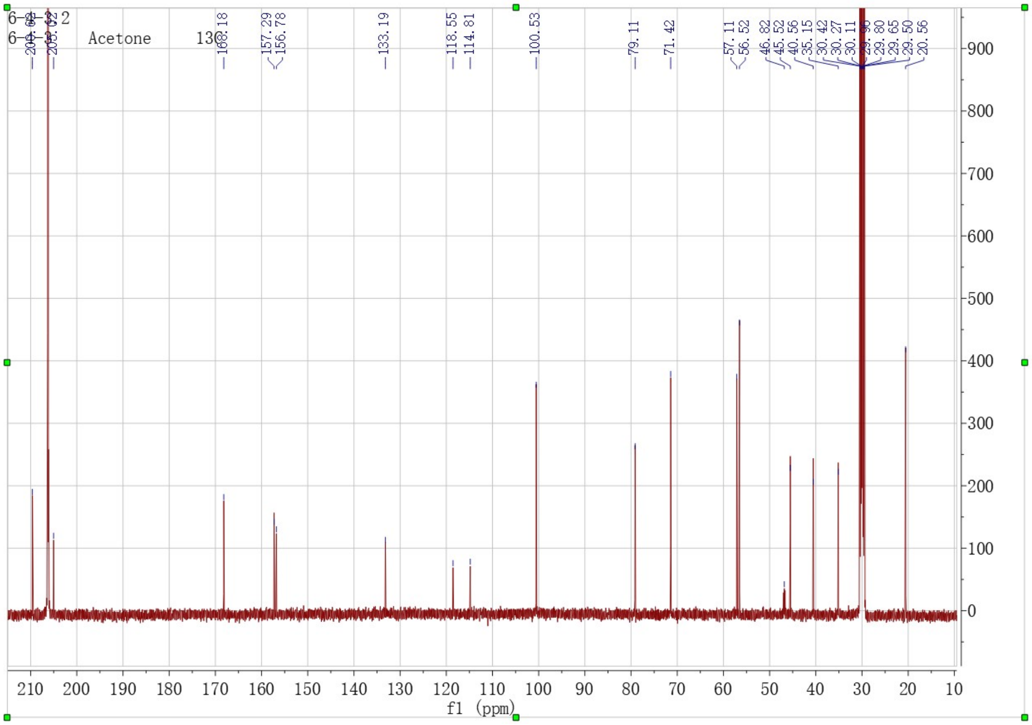


Fig. S4.2 ¹³C-NMR spectrum of compound (**5**)

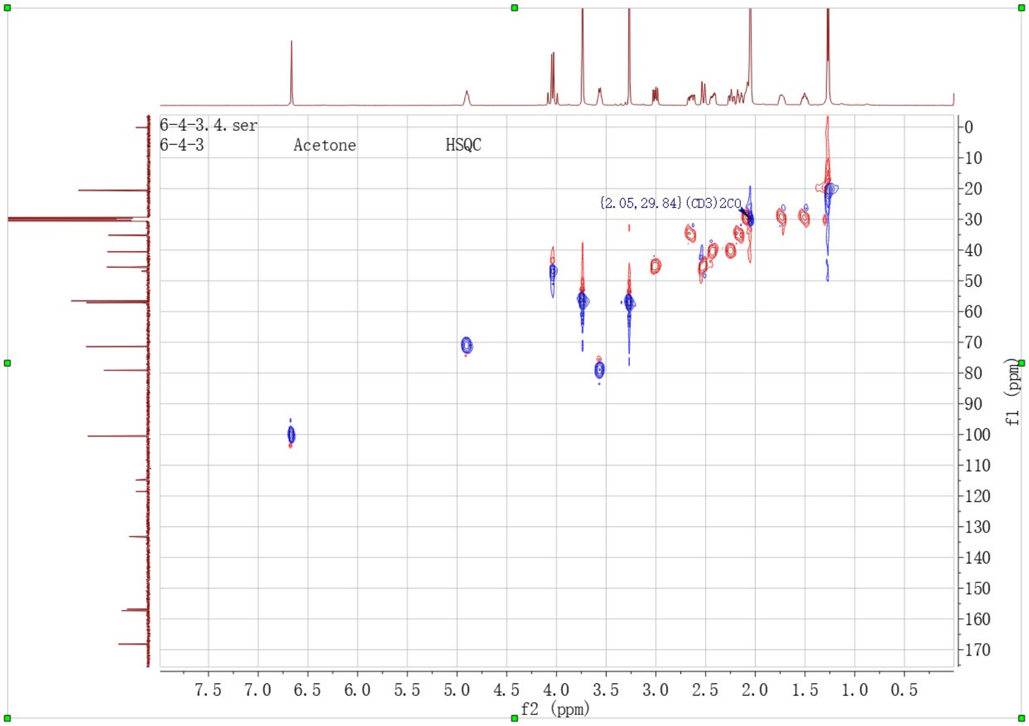


Fig. S4.3 HSQC spectrum of compound (**5**)

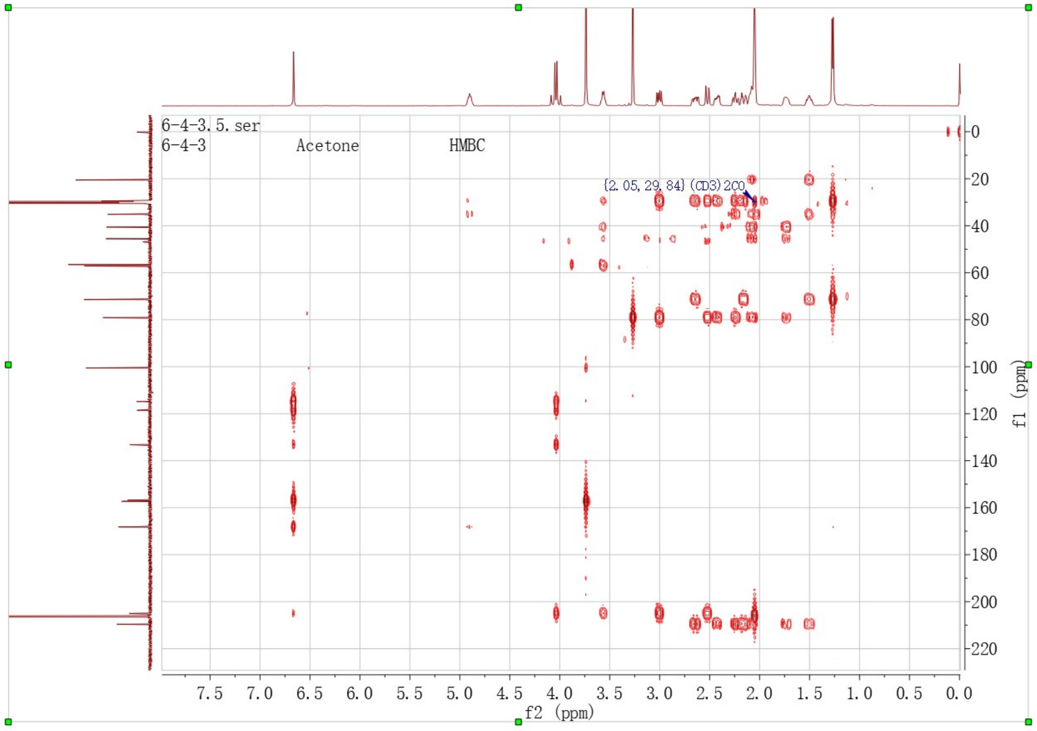


Fig. S4.4 HMBC spectrum of compound (**5**)

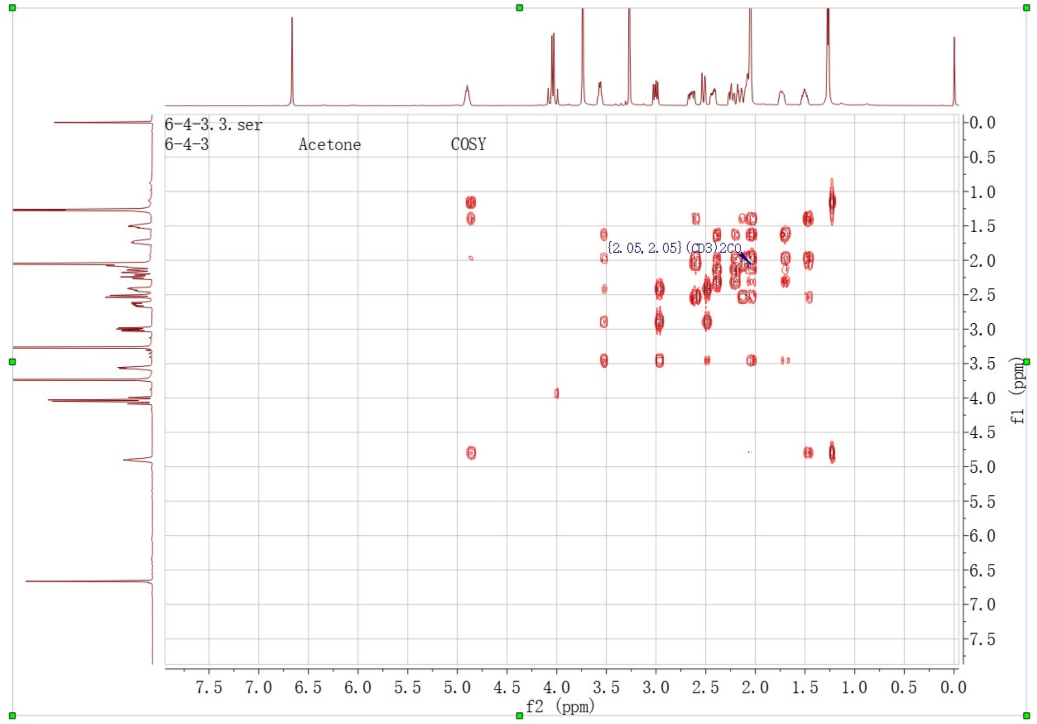


Fig. S4.5 $^{1}H$-$^{1}H$ COSY spectrum of compound (**5**)

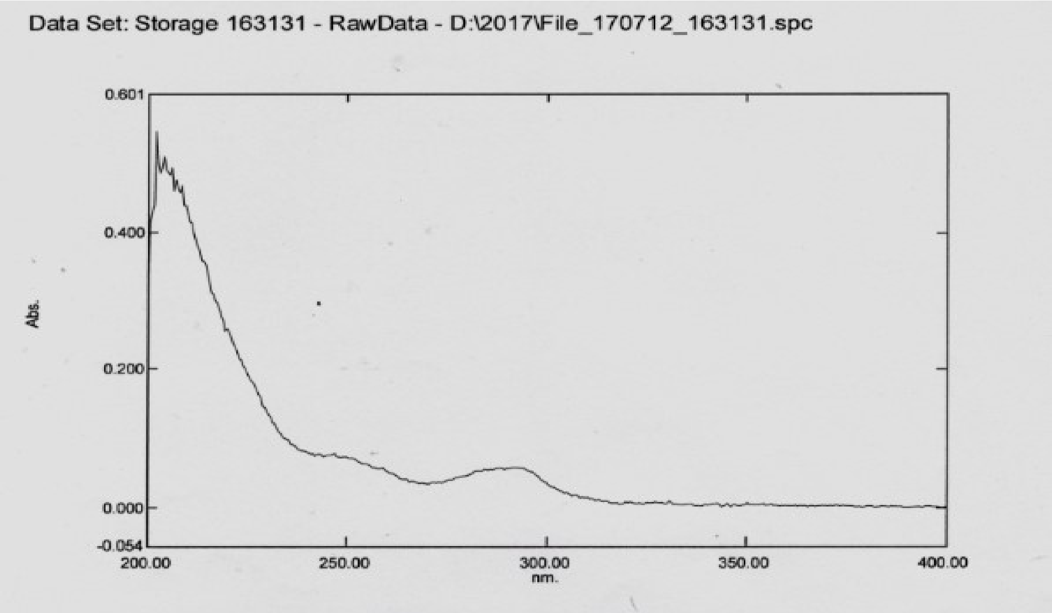


| No. | P/V | Wavelength | Abs. | Description |
|---|---|---|---|---|
| 1 | ⊕ | 291.50 | 0.058 | |
| 2 | ⊕ | 247.00 | 0.079 | |
| 3 | ⊕ | 202.00 | 0.546 | |

Fig. S4.6 UV spectrum of compound (**5**)

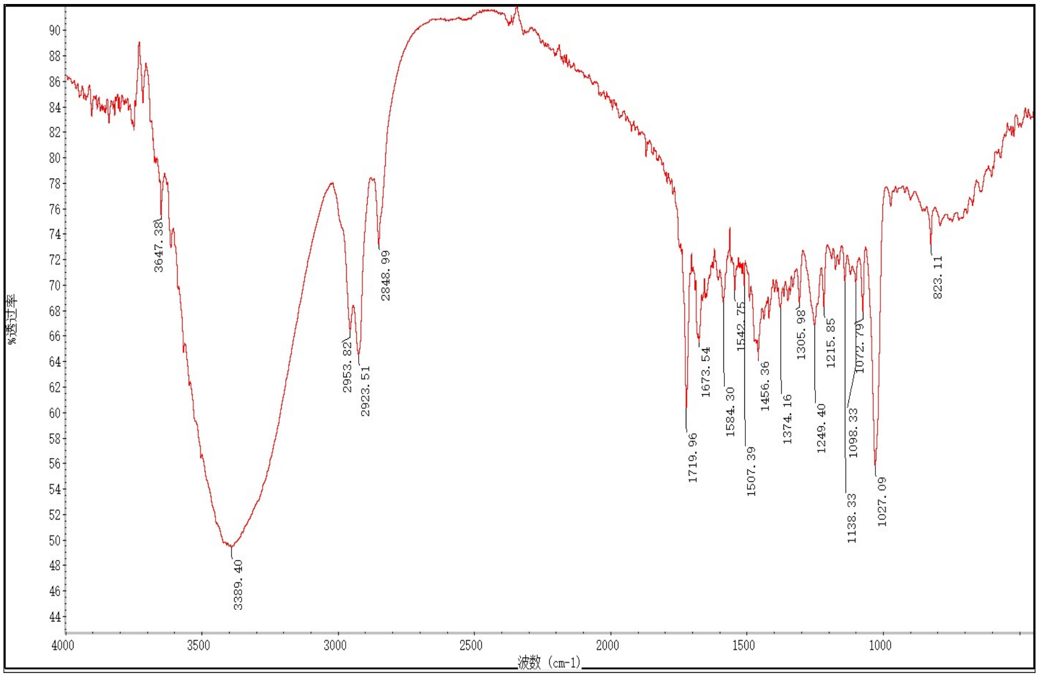


Fig. S4.7 IR spectrum of compound (**5**)

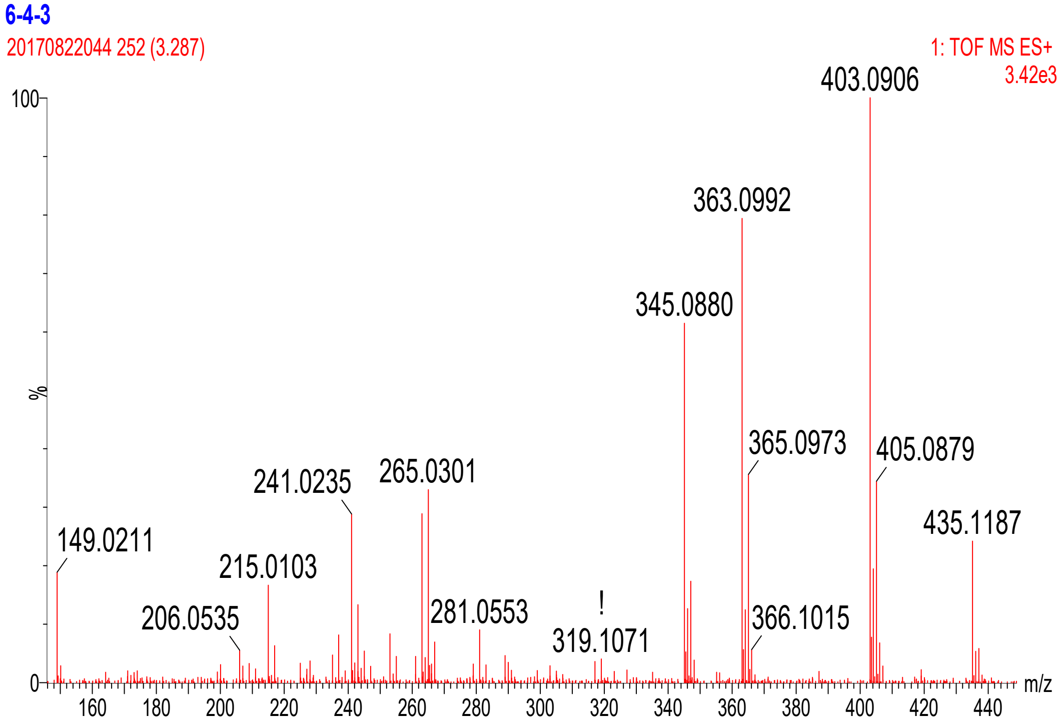


Fig. S4.8 HR-ESI-MS spectrum of compound (**5**)

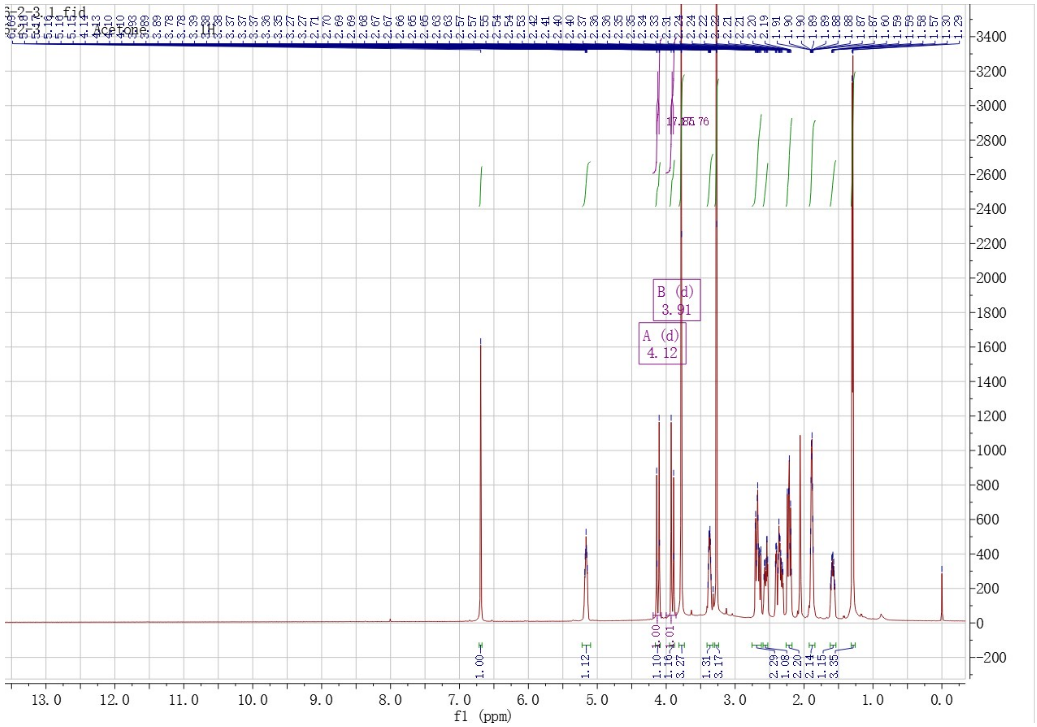


Fig. S5.1 $^{1}$H-NMR spectrum of compound (**6**)

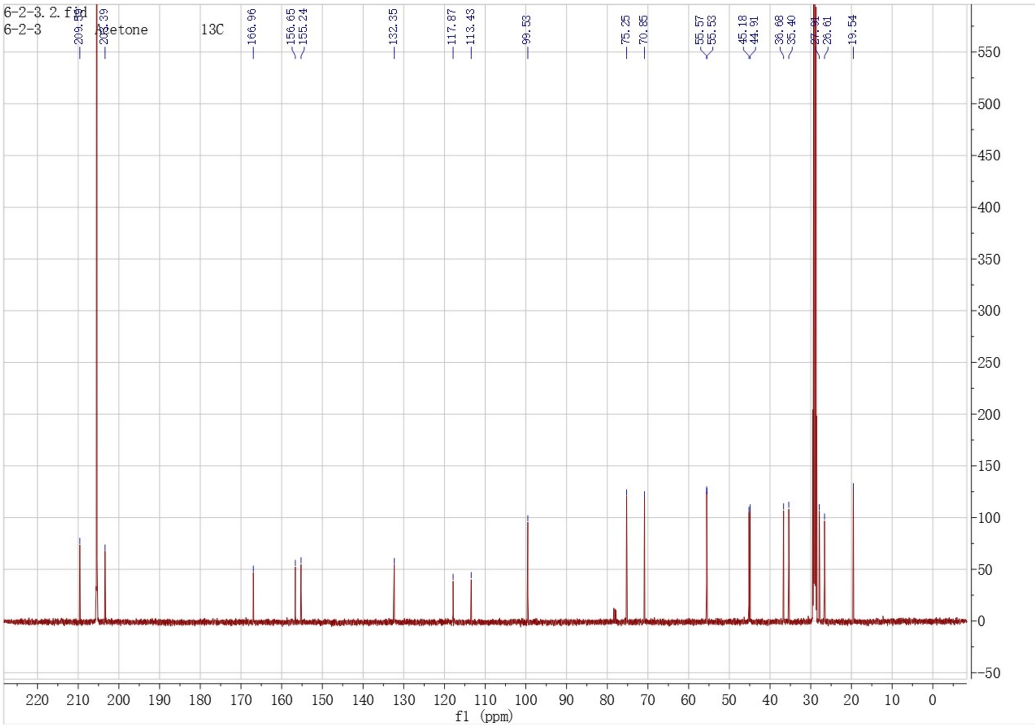

Fig. S5.2 $^{13}$C-NMR spectrum of compound (**6**)

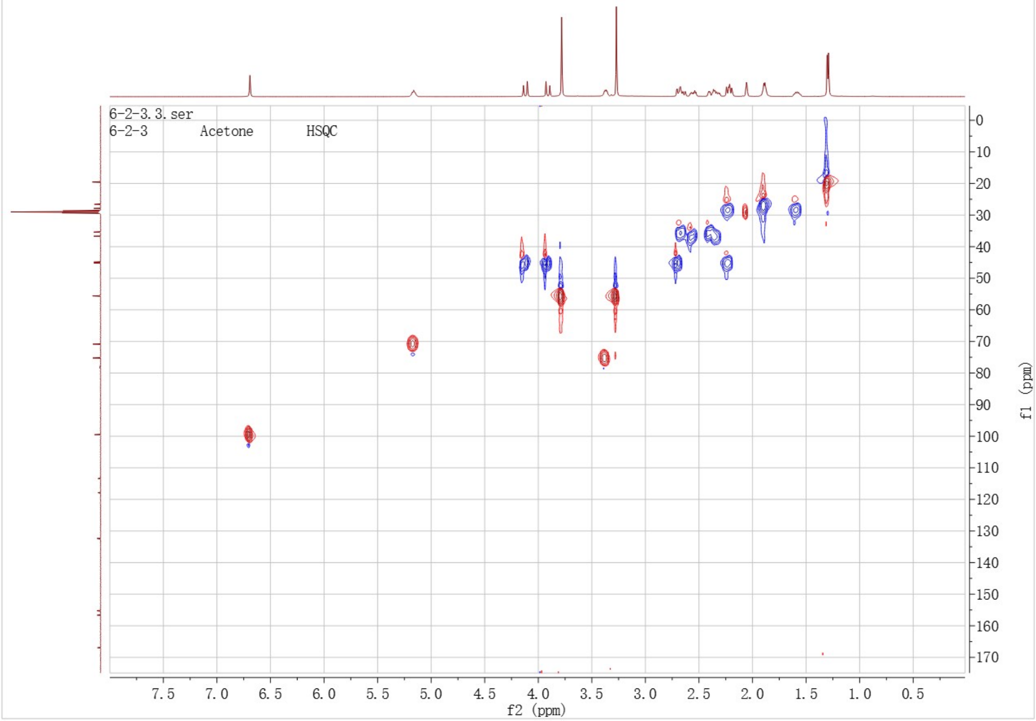

Fig. S5.3 HSQC spectrum of compound (**6**)

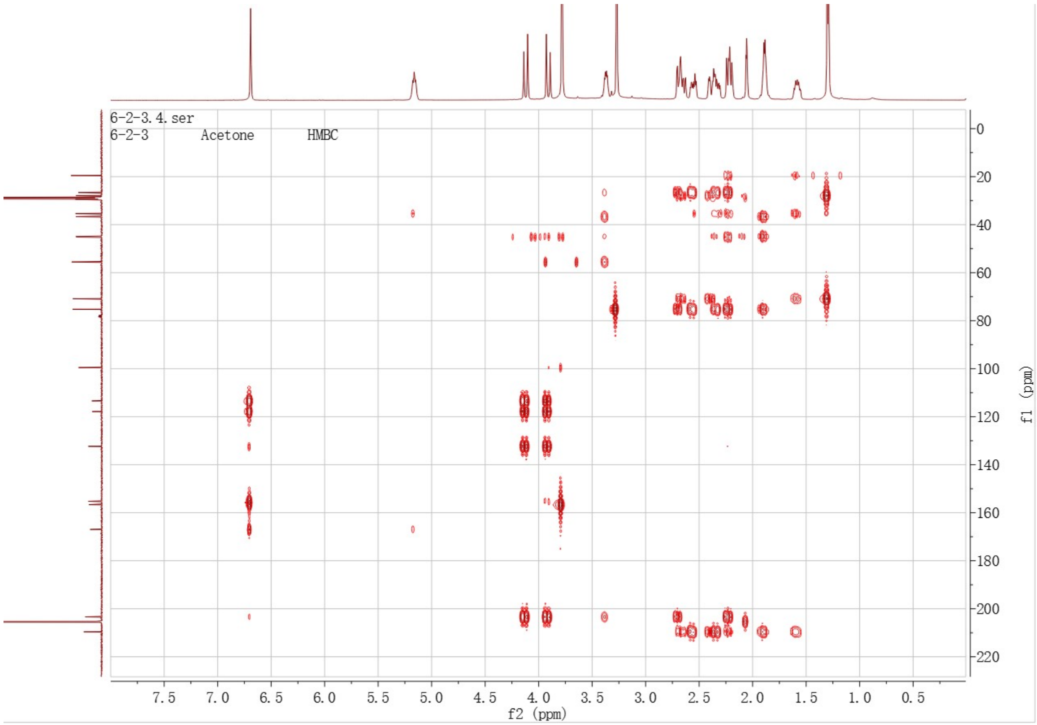


Fig. S5.4 HMBC spectrum of compound (**6**)

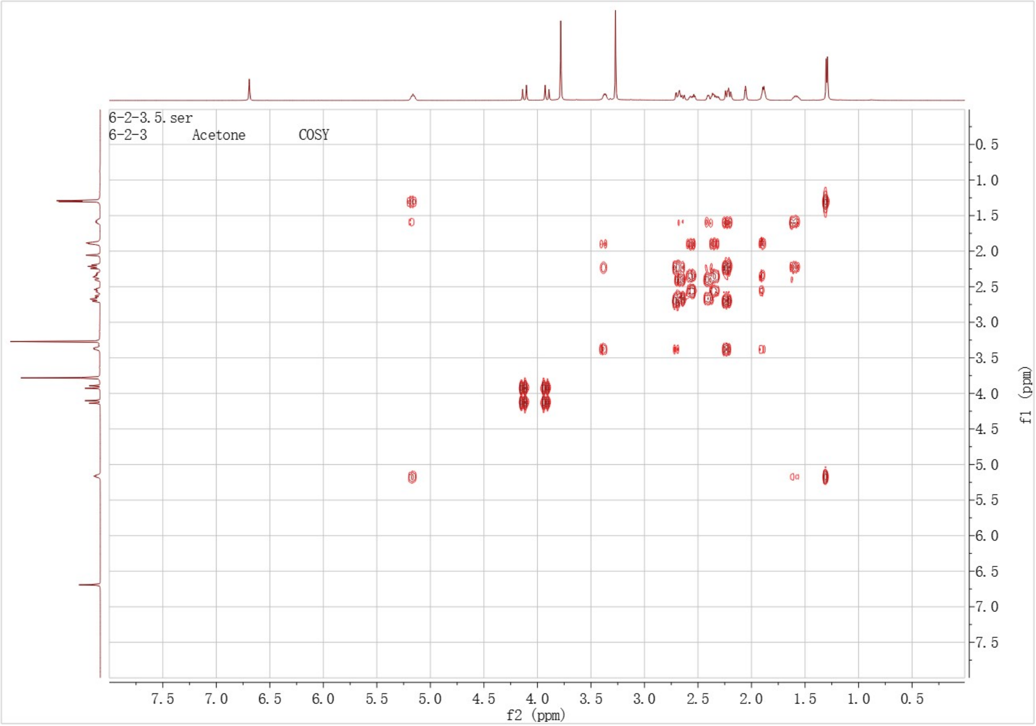


Fig. S5.5 $^{1}H$-$^{1}H$ COSY spectrum of compound (**6**)

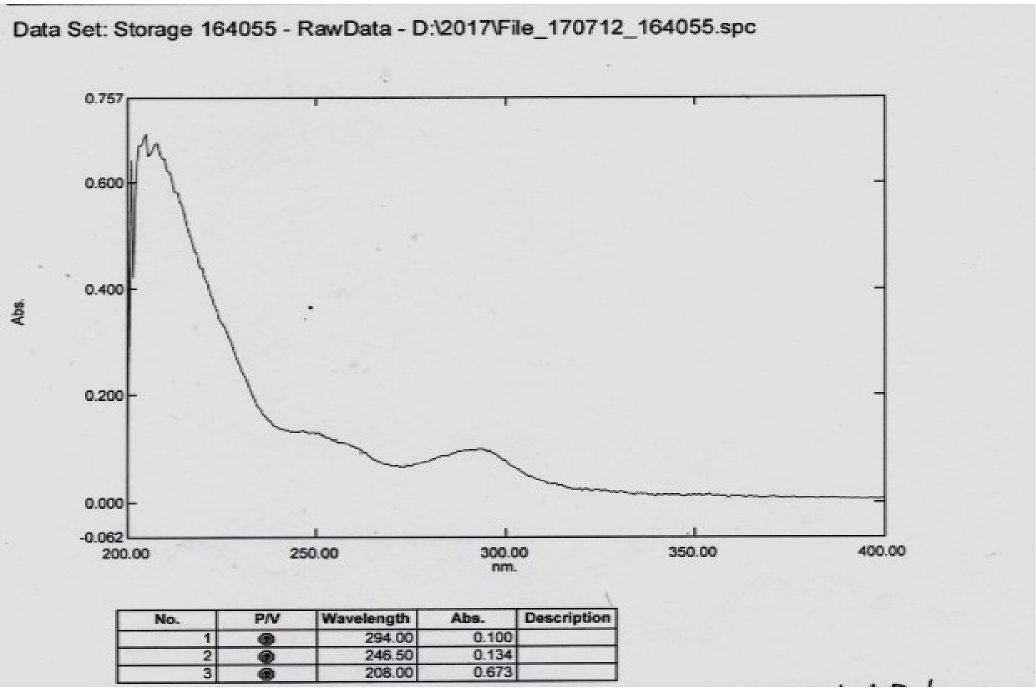


Fig. S5.6 UV spectrum of compound (**6**)

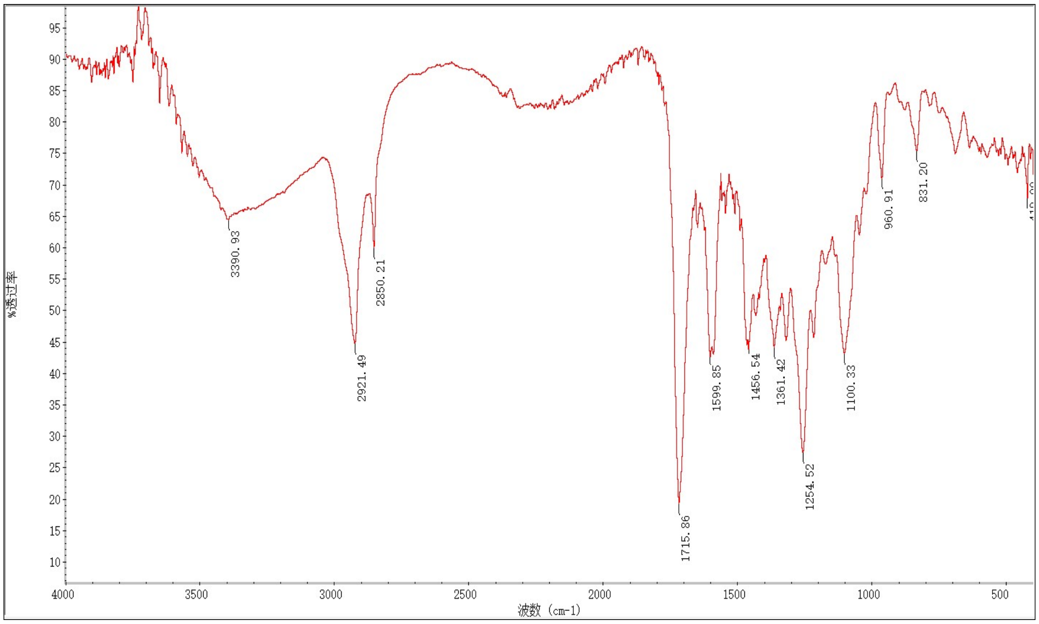


Fig. S5.7 IR spectrum of compound (**6**)

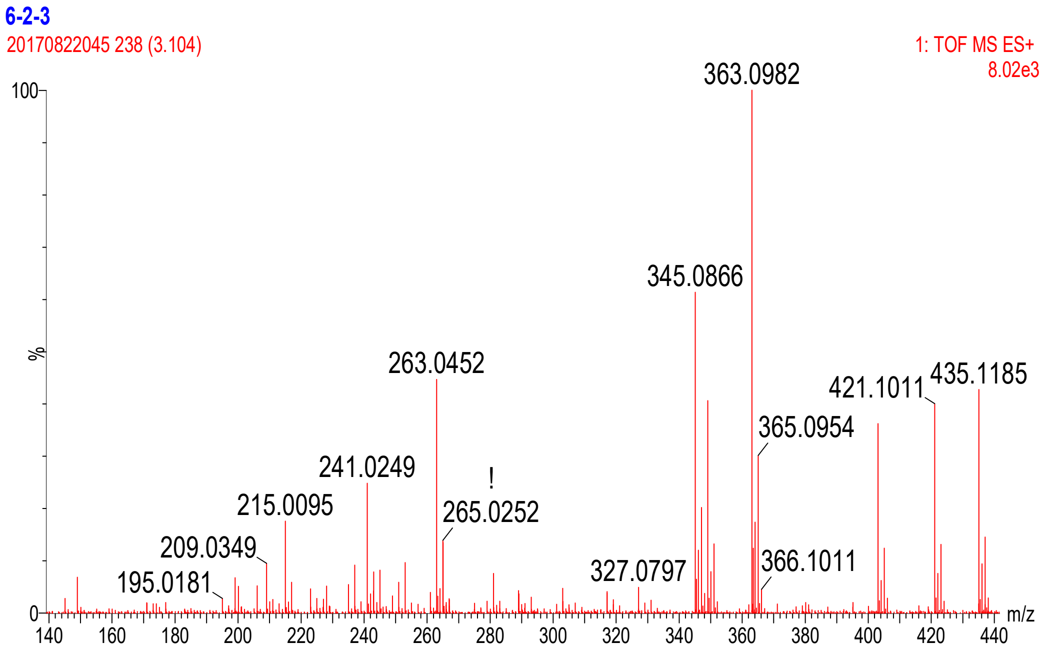


Fig. S5.8 HR-ESI-MS spectrum of compound (**6**)

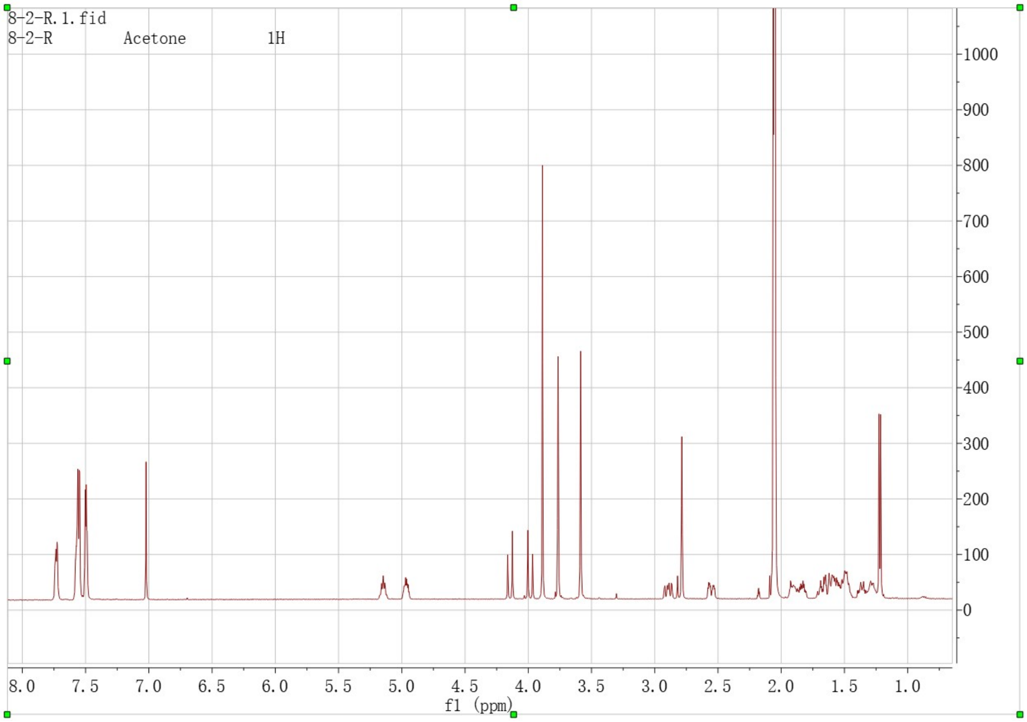


Fig. S6.1 $^{1}$H NMR spectrum (600 MHz) of **2a** in Acetone-$d_6$

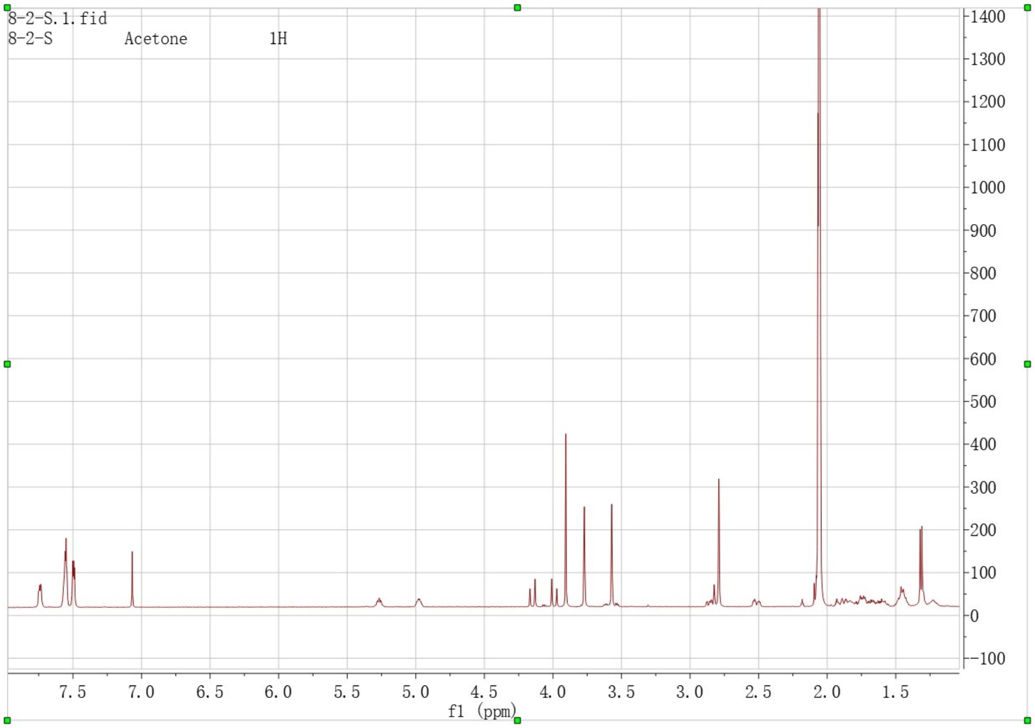


Fig. S6.2 $^{1}H$ NMR spectrum (600 MHz) of **2b** in Acetone- $d_6$

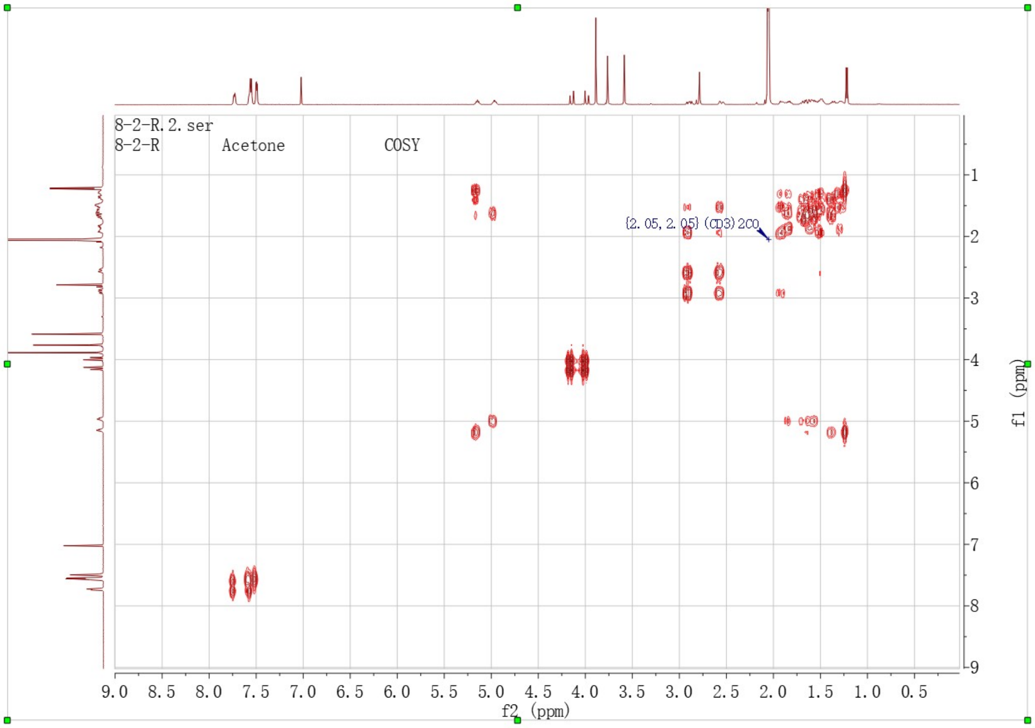


Fig. S6.3 $^{1}H$-$^{1}H$ COSY spectrum (600 MHz) of **2a** in Acetone-$d_6$

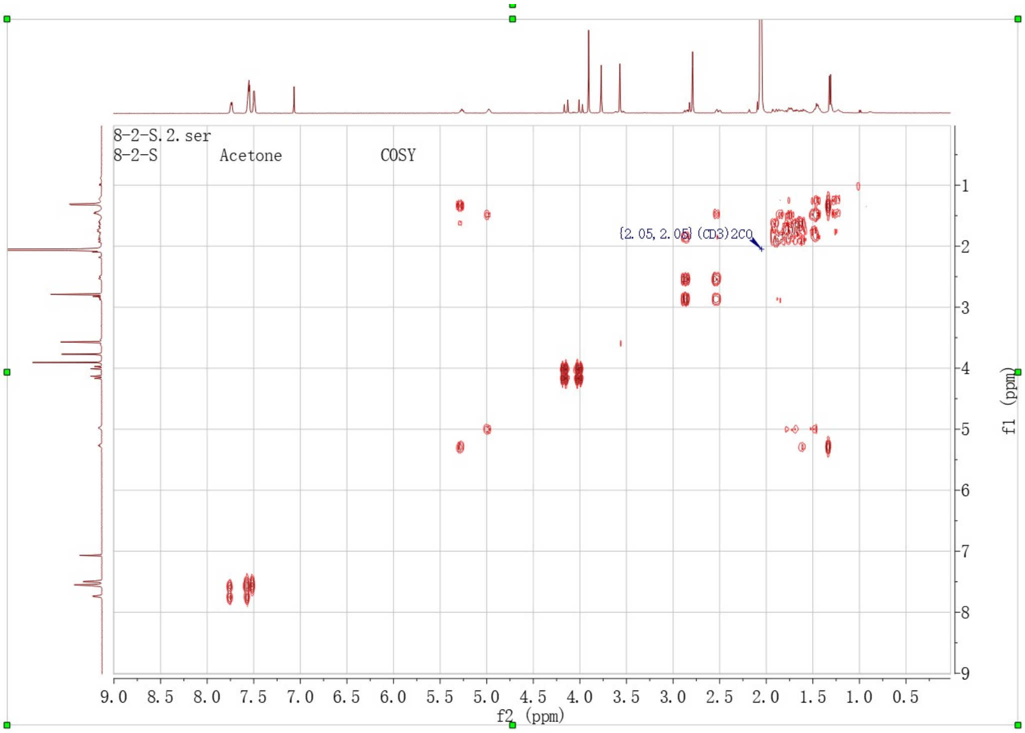


Fig. S6.4 $^{1}H$-$^{1}H$ COSY spectrum (600 MHz) of **2b** in Acetone-$d_6$

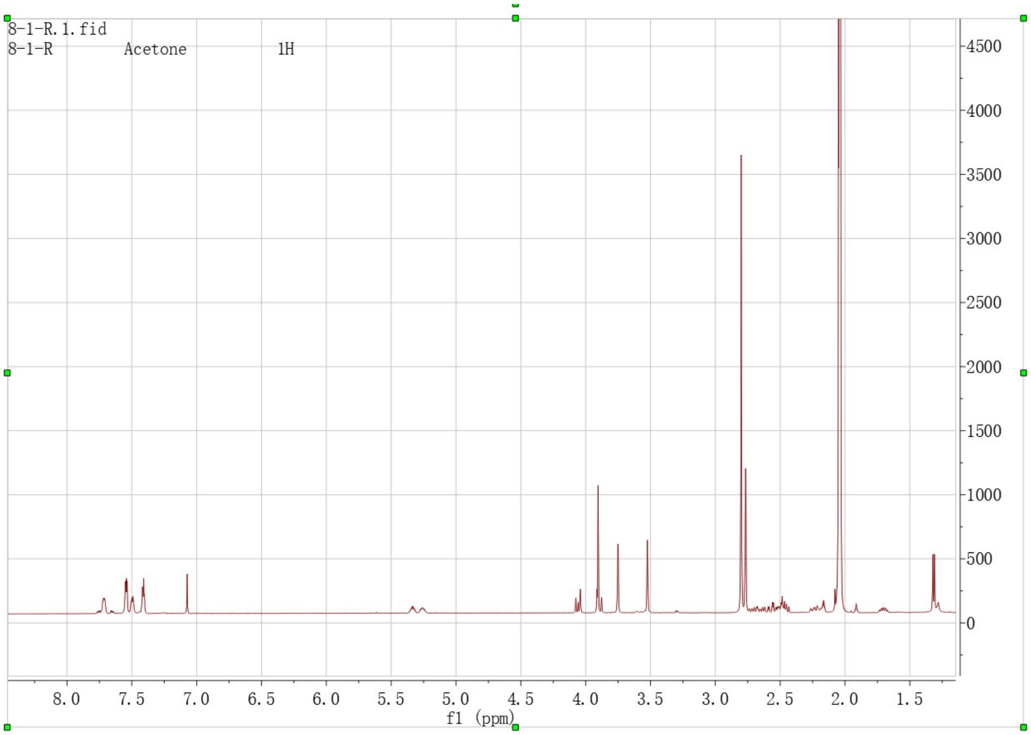


Fig. S7.1 $^{1}H$ NMR spectrum (600 MHz) of **4a** in Acetone-$d_6$

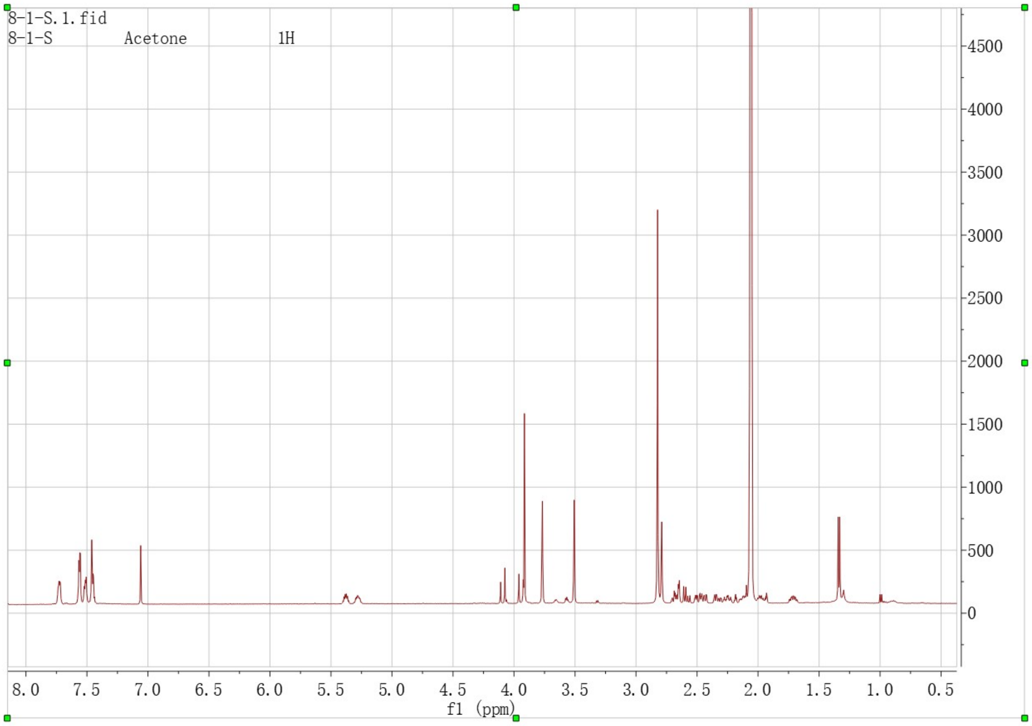


Fig. S7.2 $^{1}$H NMR spectrum (600 MHz) of **4b** in Acetone-$d_6$

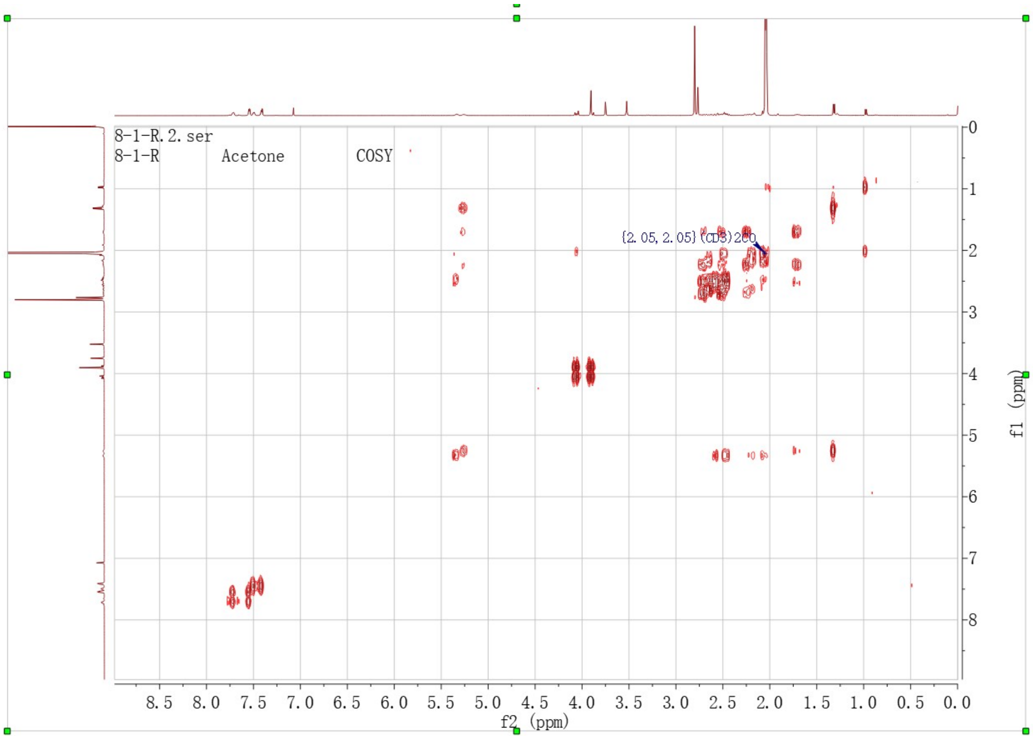


Fig. S7.3 $^{1}$H-$^{1}$H COSY spectrum (600 MHz) of **4a** in Acetone-$d_6$

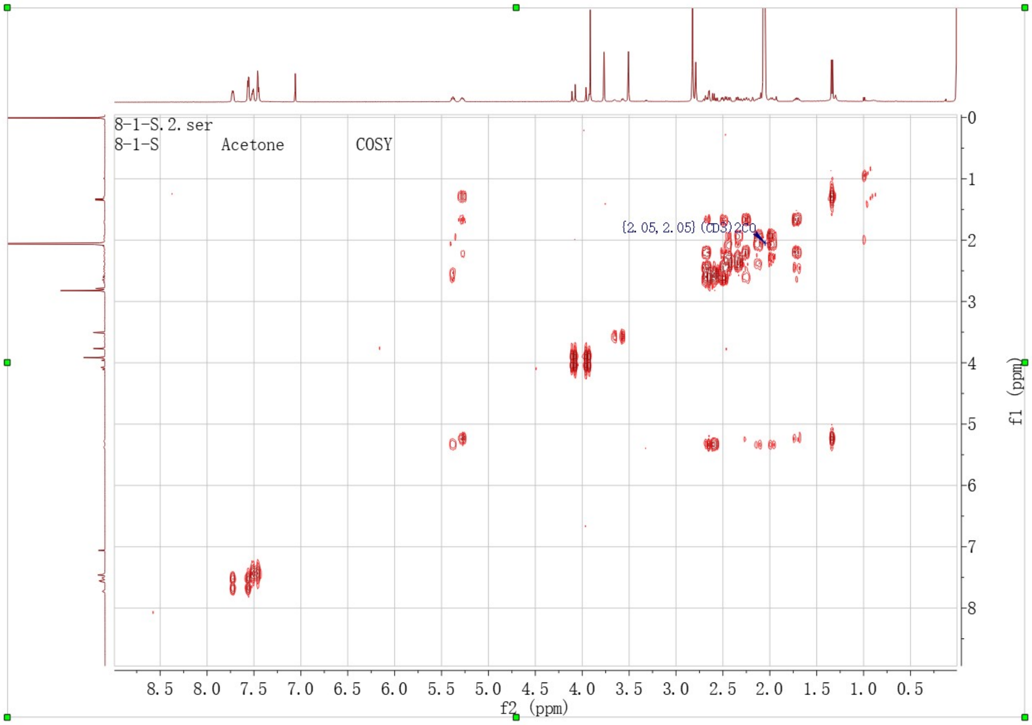


Fig. S7.4 $^{1}$H-$^{1}$H COSY spectrum (600 MHz) of **4b** in Acetone-$d_6$

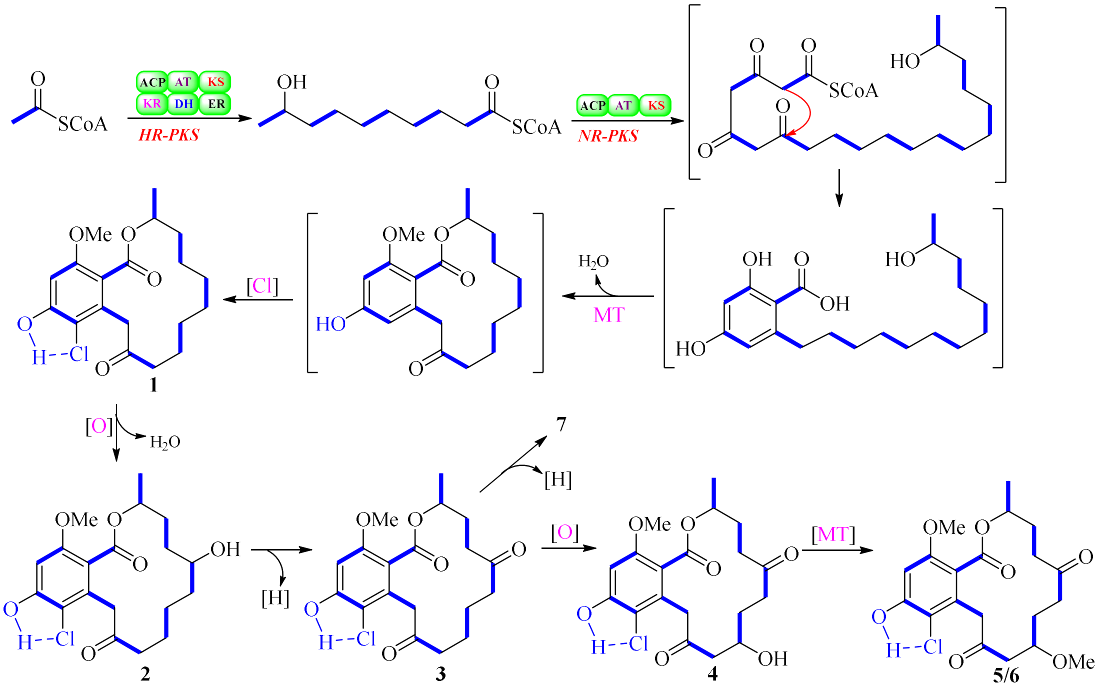


Fig. S8.1 The possible biosynthesis of **1-7**.
